# Cloud-Based Automated Design and Additive Manufacturing: A Usage Data-Enabled Paradigm Shift

**DOI:** 10.3390/s151229905

**Published:** 2015-12-19

**Authors:** Dirk Lehmhus, Thorsten Wuest, Stefan Wellsandt, Stefan Bosse, Toshiya Kaihara, Klaus-Dieter Thoben, Matthias Busse

**Affiliations:** 1 ISIS Sensorial Materials Scientific Centre, University of Bremen, Bibliothekstraße 1, 28359 Bremen, Germany; dirk.lehmhus@uni-bremen.de (D.L.); sbosse@uni-bremen.de (S.B.); 2 Industrial and Management Systems Engineering Department, Benjamin M. Statler College of Engineering and Mineral Resources, West Virginia University, 333-A Mineral Resource BLDG, Morgantown, WV 26506, USA; 3 BIBA—Bremer Institut für Produktion und Logistik GmbH, Hochschulring 20, Bremen 28359, Germany; wel@biba.uni-bremen.de (S.W.); tho@biba.uni-bremen.de (K.-D.T.); 4 Faculty of Mathematics & Computer Science, University of Bremen, Robert Hooke Str. 5, 28359 Bremen, Germany; 5 Kobe University, Graduate School of Systems Informatics, Department of Systems Sciences, 1-1 Rokkodai, Nada, Kobe 657-8501, Japan; kaihara@kobe-u.ac.jp; 6 Faculty of Production Engineering, University of Bremen, Badgasteiner Straße 1, 28359 Bremen, Germany; 7 Fraunhofer Institute for Manufacturing Technology and Advanced Materials, Wiener Straße 12, 28359 Bremen, Germany; matthias.busse@ifam.fraunhofer.de

**Keywords:** sensor integration, PEID, PLM, additive manufacturing, cloud-based manufacturing, engineering design, product customization, product design, product development, automated design

## Abstract

Integration of sensors into various kinds of products and machines provides access to in-depth usage information as basis for product optimization. Presently, this large potential for more user-friendly and efficient products is not being realized because (a) sensor integration and thus usage information is not available on a large scale and (b) product optimization requires considerable efforts in terms of manpower and adaptation of production equipment. However, with the advent of cloud-based services and highly flexible additive manufacturing techniques, these obstacles are currently crumbling away at rapid pace. The present study explores the state of the art in gathering and evaluating product usage and life cycle data, additive manufacturing and sensor integration, automated design and cloud-based services in manufacturing. By joining and extrapolating development trends in these areas, it delimits the foundations of a manufacturing concept that will allow continuous and economically viable product optimization on a general, user group or individual user level. This projection is checked against three different application scenarios, each of which stresses different aspects of the underlying holistic concept. The following discussion identifies critical issues and research needs by adopting the relevant stakeholder perspectives.

## 1. Introduction

Imagine there’s a product that starts collecting data from the moment that it’s being made—a product that collects, then evaluates these data, and passes the results on into the cloud: information that describes its making and its everyday experiences. In the cloud, this evidence is matched with what other individual products of identical type and make provide, and scrutinized on an item and a type level. In the cloud, this information is processed to automatically adapt design and dimensioning of coming product generations. But why wait until a coming generation? And, why, specifically, do so if all the information needed is readily at hand to optimize the product not only for some general set of requirements, as sophisticated as they may be, but for an individual customer, and an individual use pattern? Why not allow the product to evolve, not from generation to generation, but from item to item? And do so differently from individual customer to individual customer, too?

**Figure 1:**
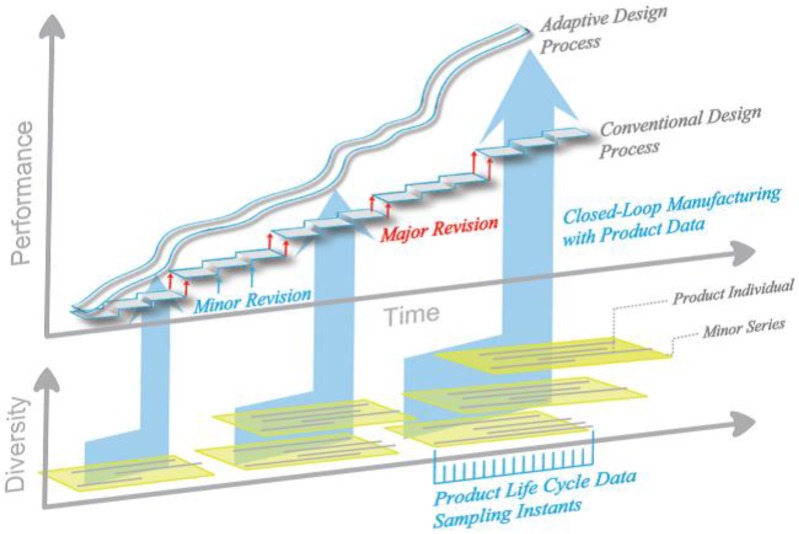
Consequences of the basic concept visualized: From product generations to continuous optimization through gathering and usage of life cycle data in conjunction with flexible production.

Figure 1 graphically represents the impact of this concept on product generations and diversity, with continuous change replacing the major revisions (these are retained in the new approach on lower level with e.g., feature addition as discriminator, leading to product groups with close internal and more remote external relation) as well as the minor ones of a more common organization of the process. Conventional production technology, specifically in mass production, is not matched easily with this vision. Mass production typically relies on complex manufacturing equipment and thus high investment costs to reduce part costs based on an economy of scales approach: Flexibility is sacrificed for the sake of productivity. Tools are matched to products, causing adaptation of the latter to be costly. What, then, if production processes provided boundless flexibility, and changes to the product could be realized virtually, as changes to its digital representation, and at virtually no cost?

In a manufacturing environment of this kind, the making of the product would not be linked to the physical site at which dedicated tools and machinery were kept, simply because no such tools and machinery would be needed. Instead, availability of the digital product information alone would enable countless manufacturing centers worldwide to create the product without lead-time. This is why such approaches have been termed Direct Digital Manufacturing [1].

A global manufacturing environment like this could transform the way we make things. The paradigm shifts implied are manifold: For one thing, as product development would not require parallel development of production equipment, design and production could move further apart. At the same time, and as described above, flexibility in manufacturing could be used to optimize products on a customer or customer group basis, and furthermore, to implement a continuous design optimization. Practically this means nothing less than brushing aside the fundamental concept of individual product generations. Since optimization needs a basis, a primary prerequisite is a usage data link allowing backflow of information from product to designer. Equally important is a legally and technically secure access for the various potential producers and service providers to initial and optimized product design and manufacturing information. This global access to (general product and usage) data, software tools and finally manufacturing resources is the primary link of our scenario to the concept of the cloud (please consider also Figure 8 in Section 3 in this respect). Cloud-based manufacturing (CBM), sometimes also designated Cloud Manufacturing (CMfg), in general is currently the object of intense study [2,3,4,5] Xu *et al.*, to give an example, introduced cloud manufacturing via the earlier-adopted concept of cloud computing, defined by the National Institute of Standards and Technology (NIST) as “a model for enabling ubiquitous, convenient, on-demand network access to a shared pool of configurable computing resources (e.g., networks, servers, storage, applications, and services) that can be rapidly provisioned and released with minimal management effort or service provider interaction.’’ [6]. Cloud manufacturing extends Cloud Computing by including production processes (scheduling, resource planning *etc.*) and related Cyber-Physical Systems (CPS) as active units [3]. Another definition has been provided by Wu *et al.*, who discuss whether CBM is indeed the paradigm change as which it is currently being advertised. Their answer is in the affirmative: By reviewing the suggested definitions of the field and adding their own perspective, they manage to delimit CBM both from earlier concepts like flexible and redistributable manufacturing systems and intermediate development stages like web- and agent-based manufacturing (WBM, ABM) [7]. A true CBM approach, in their eyes, needs to integrate “Infrastructure-as-a-Service (IaaS), Platform-as-a-Service (PaaS), Hardware-as-a-Service (HaaS), and Software-as-a-Service (SaaS)” elements and is distinguished from WBM and ABM by its capability of facilitating new business models through such elements [7,8]. In further publications, Wu *et al.* have linked their studies to additive manufacturing and looked at product design, too, thus extending the original term CBM to cloud-based design and manufacturing (CBDM) [9,10]. Interestingly, however, the usage data feedback is not considered a core feature of CBM in these definitions: Data from the usage or Middle-of-Life (MoL) phase is included in some of the projections offered, though not in an automated fashion, but solely by way of direct end user (customer) feedback and integration in the design process (customer co-design). The practical implementation of such end user-centered feedback facilities is seen as major research issue. Besides, as we will show later in case study 2 (Section 4.2), the notion of separated ABM and CBM approaches brought forward by Wu *et al.* can be overcome [9,10].

All the aforementioned capabilities require sophisticated analysis of data as glue between the various enablers on technological level. On a more generic level, Artificial Intelligence (AI), Machine Learning (ML) and advanced ICT are already integrated in most areas of daily life, as well as in industrial production and product development. In parallel, the value of information and data is rapidly increasing—more so if they help in satisfying customer needs. Companies who understand the needs of their customers and at the same time are capable of translating them in a timely manner into new products and product enhancements, have a competitive advantage in the global business environment.

This statement accepted, several equally important aspects are required to succeed in the future:

- Availability of information about the usage of individual product items.
- Capability to analyze and translate this information into technical requirements.
- Capability to map these requirements to product design (e.g., CAD model).
- Capability to economically manufacture the products designed accordingly.
- Capability to increasingly automate these steps to realize fast time-to-market.

**Figure 2:**
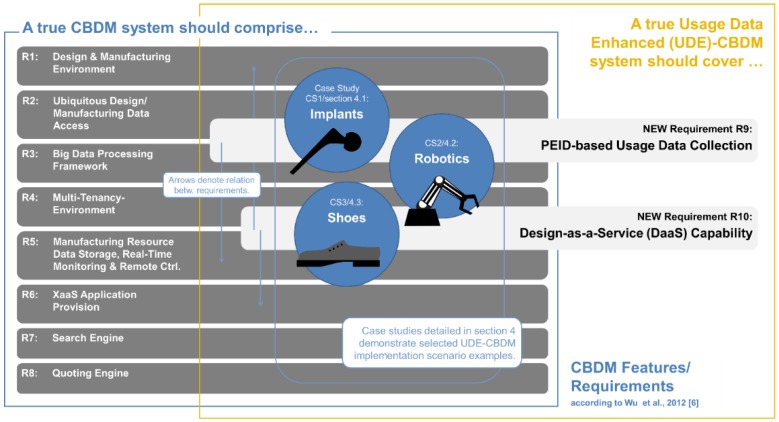
Requirements or criteria which characterize a CBDM approach, and additional ones which constitute its extension to Usage Data-enhanced (UDE)-CBDM.

That said, we may match our own perspective of what might be called “Usage Data-Enhanced Cloud-based Design and Manufacturing” or UDE-CBDM to the requirements proposed in the literature to distinguish cloud-based from conventional manufacturing. We have done so in Figure 2, contrasting the set of eight requirements a CBDM approach has to meet according to Wu *et al*. [8] with two additional requirements that together with the initial ones constitute the UDE-CBDM case.

The additions we suggest are defined as follows:

Requirement 9, PEID-based Usage Data Collection, requires that perceptive product usage data should continuously be collected and stored in a cloud-based data repository.

Requirement 10, Design-as-a-Service (DaaS) Capability, requires that a cloud-based service and the associated tools should be established that retrieves data from the aforementioned repository and transforms it into an adaptation of the products original root design that reflects the usage information contained in the data by improving product performance, economy or the like on this basis.

The reader should note that these are minimum requirements constituting a UDE-CBDM scenario. We have deliberately dispensed with including AM or automated design approaches on this level—ultimately, we believe these techniques to be a necessity (in case of AM) or a logical development (automated design)—but they can still be omitted in a formal definition, as solutions that do not build on them can theoretically be envisaged.

In practical implementations of this framework, given that (consumer and professional) products are often used in a rather unique way by the customers, the usage information obtained is most likely on an item-level. Today’s research in Product Lifecycle Management (PLM) is focusing on the rather complex problem of how to capture, organize and present information along the complete lifecycle. In the vision of this paper, PLM is the conceptual foundation providing the necessary information to the other processes, e.g., from usage to design and manufacturing. On a technological level, combinations of products with so-called product-embedded information devices (PEIDs) are required. PEIDs can be seen as smart sensor systems or cyber-physical systems (CPS, which brings the topic of their integration into products down to the field of sensor integration and material-integrated intelligent systems.

Once the information is obtained and analyzed, which is far from trivial [11,12], the design and manufacturing of highly individualized products adds further complexity. Whereas one-of-a-kind-production and similar concepts have been discussed for years, today’s advanced tools incorporate the opportunity to automate many of the time-consuming steps involved. Knowledge-based engineering [13] and adaptive design [14] automate specific tasks in the engineering domain while additive manufacturing gives designers and manufacturers more freedom to realize products.

The only suitable manufacturing process class that may provide the flexibility our vision requires is additive manufacturing (AM), which is capable of producing a part directly from a digital model. This way, product modifications can in principle be handled entirely in the virtual world, allowing economic definition and production of individual product variants. However, this choice of a process class has implications for sensor integration, since there is the need to link both topics. Thus in our vision, additive manufacturing is important in two different ways, firstly, regarding the aforementioned degrees of freedom during production and, secondly, by facilitating the integration of the necessary sensors to capture usage information. Additive manufacturing thus becomes the link that closes the loop: The capturing of information during the usage phase is a prerequisite for the whole vision to work, and sensors are required to obtain this information, even though complementary information can be collected from other sources as well [15].

With this overall vision in mind, the different areas and their current state of the art are illustrated in the second chapter. In its first Section 2.1, product life cycle management (PLM) and item-level information flows, as well as Intelligent Products as one way of capturing information, are introduced. Following this, in Section 2.2 the focus is put specifically on product usage information, based on its key role in our concept, before the topic of sensor-integration in additive manufacturing is touched upon in Section 2.3. Within it, additive manufacturing is described in greater detail, looking into the current ability to create complex and multi-material structures. Section 2.4 explores how automated design, based on PLM data, may be capable of creating customized products in an efficient and timely way. The synthesis of all elements contributing to our concept is explained in more detail, though still on a level of principles, in Section 3. It is further illustrated via three comprehensive case studies, which together form Section 4. The first of these is focused on medical implants. In this application, the generation-to-generation adaptation based on strictly individual use patterns is a challenge that links up to our vision especially in terms of the need for customization. Besides, the application as such is already associated to AM, which is an established manufacturing process in this field, and to sensor integration, though in the latter case mainly on an academic level. The second case addresses robots as products that are integrated in a closed-loop adaptive design and manufacturing process. They contribute their inherent connectivity and sensor integration to support the cloud-based manufacturing vision. The third case represents a near-complete implementation study of our vision, using the shoe industry as example. Except for an extensive automation of the usage data-driven design processes, all elements of the present study’s underlying notions are reflected in this example.All case studies are critically discussed in Section 5, alternately assuming the positions of the various stakeholders involved. Section 6 concludes our paper and provides an outlook on further paths of research in the area.

## 2. Fundamental Technologies and Concepts: A Literature Review

The vision we propose is covering an exceedingly broad spectrum of technologies, ranging from Additive Manufacturing (AM) via Product Life Cycle Management (PLM) to existing concepts of Cloud-based Design and Manufacturing (CBDM) and sensor integration. In the present section, the state of the art of such enabling technologies is illustrated, providing a solid, common foundations for our readers, whom we expect to come from several different backgrounds. The aim is to allow all of them, irrespective from which research community they may stem, to acquire basic knowledge in adjacent fields which will help them grasp the implications and potential of the framework we propose, and follow the arguments in the case studies and discussion sections more easily. Note that we have refrained from covering CBDM in this context, since we consider this the broader basis and have already introduced it as such in Section 1.

### 2.1. Product Lifecycle Management (PLM)

**Figure 3:**
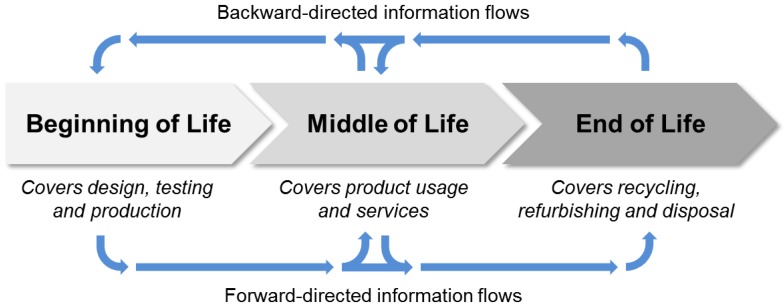
Model of a product lifecycle and important information flows [18].

PLM is the process of handling product data, information and knowledge across a product’s lifecycle [16]. The terms “data” and “information” are used synonymously in the following for simplicity’s sake. The product lifecycle is a concept lent from biology where it describes the recurring change of states for certain organisms (see [17] for biological lifecycle). In engineering, the organisms are exchanged for tangible goods (products) and the state changes are substituted by processes, such as design, production, use, repair, recycling and disposal. Since lifecycle processes differ among the targeted products, they are generalized into three phases stated as “beginning of life” (BOL), “middle of life” (MOL) and “end of life” (EOL) as illustrated in Figure 3. The BOL covers the design and the realization of the product, the MOL concerns the product’s usage and related value-adding services, while the EOL typically consists of several optional activities, such as reuse by other customers (second hand), remanufacturing (refurbish used product), material recycling and disposal. Along the product lifecycle, matter, energy and information is exchanged and transformed. Multiple internal and external stakeholders are involved along the lifecycle of a product.

Traditional information in PLM focuses on product types rather than individual products, examples are 3D models and parts lists. These data are called master data—*i.e.*, data that is changing seldom. An increasing amount of information along the lifecycle is related to the usage and service of products. Typical for this information is that it describes specific items, *i.e.*, identifiable, unique products. Though there have been research projects in the past (e.g., [19,20]), the transition from traditional PLM to an item-level perspective (*i.e.*, item-level PLM) is still a grass-roots movement.

From a technical point of view, item-level PLM is conceptually related to Intelligent Products, which are also known as Smart Products [21]. These products are one possible way to enable an individual product to generate, store and communicate data throughout its lifecycle. Intelligent Products are physical items, which may be transported, processed or used and which comprise the ability to act in an intelligent (“smart”) manner of various degrees [22]. McFarlane *et al.* define the Intelligent Product as *“[...] a physical and information based representation of an item [...] which possesses a unique identification, is capable of communicating effectively with its environment, can retain or store data about itself, deploys a language to display its features, production requirements, etc., and is capable of participating in or making decisions relevant to its own destiny”* [22]. In order for intelligent products to realize the aforementioned potential, the capabilities of sensing, storing, processing and communicating information has to be implemented.

Product Embedded Information Devices (PEIDs) [23] describe a realization of the concept of Intelligent Products. They act as embedded information gathering devices linked to sensors, which are able to sense their environment and their condition and communicate the data gathered wirelessly. PEIDs are categorized according to their capabilities with regards to data storage and data processing [24]. In addition to these features, the devices’ ability to integrate sensors, as well as their options for network connectivity are used to distinguish different types of PEIDs. Since PEIDs typically contain a global, unique identifier one of the most basic requirement towards mapping the generated information to an individual product is met. Information delivered by PEIDs has to be integrated into the overall PLM system [25].

### 2.2. Product Usage Information

According to a working definition provided by Wellsandt *et al.*, “usage information” is “[…] any product-related information that is created after the product is sold to the end customer and before the product is no longer useful for a user.” [18]. There are different media providing access to usage information, such as social media, maintenance reports, customer complaints hotlines and PEIDs [26]. Since this paper focuses on Intelligent Products, only PEIDs will be considered further. PEIDs measure the current state of the product item, its environment and any relevant actors (e.g., the user). This measured raw data has to be transformed into useful information. The transformation can be a complex task, potentially involving statistics and pattern recognition. The resulting usage information describes objects, procedures and events of the MOL phase. Other information sources might be necessary to deliver contextual information in case the sensor data cannot be unambiguously understood.

**Figure 4:**
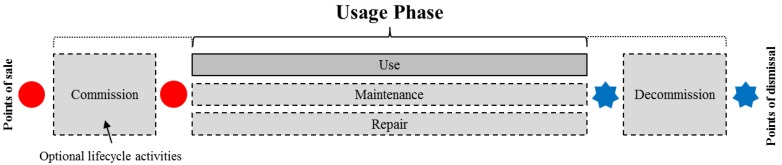
Middle of life phase for durable goods (adapted from [27], see also [26]).

In product design, information about the usage of products helps engineers to understand, for instance, which product features are fit for purpose and which ones are not. Usage information is describing different activities of the usage phase. For durable goods with a long lifetime, the MOL phase might consist of five distinct processes as illustrated in Figure 4.

The figure concerns a single user scenario only. It consists of the actual usage activity and four optional supplementary activities. Optional activities are can be relevant for some types of product but not for others. The first process that is conducted in the MOL phase is the commission of the delivered product item. During commission, the product is prepared for the actual usage by, for instance, connecting it to local infrastructure, configuring its software and adjusting it to meet the personal characteristics of the user. Commission can be done by the user or a service provider. The following *use* activity provides the actual benefits of the product to its user. It can be interrupted by *maintenance* activities aiming to preserve the functions of the product. In case the product is broken, *repair* activities can be performed. When the product item is no longer useful for the user, it is decommissioned. The decommissioning of the product can be conducted by the user or a service provider. In case the commission and decommission activities are conducted by third parties, the activities do not belong to the middle of life in a formal sense. The information gathered during these activities might be, however, still considered usage information.

### 2.3. Additive Manufacturing Technology and State of the Art

Additive Manufacturing is the physical basis of our concept, which sinks or swims with the almost boundless flexibility AM promises—and actually delivers in most respects. Nevertheless the concept comprises requirements for features that surmount what the state of the art has to offer. Primarily, this concerns the ability to easily include sensors, intelligent sensor systems or PEIDs in the building job. The present chapter will approach these issues step by step, in the course providing an overview of current AM capabilities.

#### 2.3.1. Processes and Business Cases for Conventional AM

The game-changing potential of Additive Manufacturing has been spelled out by Campbell *et al.* in a recent foresight study: “Designs, not products, would move around the world as digital files to be printed anywhere by any printer that can meet the design parameters. The Internet first eliminated distance as a factor in moving information and now AM eliminates it for the material world. Just as a written document can be emailed as a PDF and printed in 2D, an ‘STL’ design file can be sent instantly to the other side of the planet via the Internet and printed in 3D” [28].

As technological area, Additive Manufacturing (AM) bears a multitude of names, some of them misleading. There is, however, a common definition that allows a clear distinction between what is in fact AM, and what is not: According to ASTM international committee F42, Additive Manufacturing is the “process of joining materials to make objects from 3D model data, usually layer upon layer, as opposed to subtractive manufacturing methodologies” [29].

Table 1 provides a rough overview of available additive manufacturing processes and links them to the material classes processed. Where process and material intersect, concrete examples and references explaining them in more detail are provided. The list is not conclusive, but provides examples and associated references only.

**Table 1:** Examples of AM techniques available today, classified by basic principle according to ASTM F2792-12a [29] and class of materials.

Basic Principle	Class of Material		
	Polymer/Organic	Metal	Ceramic
Binder jetting		3D printing ^1^ [30]	3D printing ^1^ [31,32]
Directed energy deposition		Laser Engineered Net Shaping/LENS™ [33], Directed Light Fabrication/DLF [34], Direct Metal Deposition/DMD [35]	Laser Engineered Net Shaping/LENS^TM^ [36]
Material extrusion	Fused Deposition Modelling, FDM [37]	Fused Deposition Modelling, FDM [38,39]; Multiphase Jet Solidification, MJS [40]	Fused Deposition Modelling, FDM [41]; Robocasting [42]; Freeze-form Extrusion Fabrication, FEF [43,44]
Material jetting	Direct Printing, DIP [45]; Multi-Jet Modeling/MJM or Polyjet Modeling/PJM [46].	Direct Printing, DIP [47]	Direct Printing, DIP [32]
Powder bed fusion	Selective Laser Sintering, SLS ^2^ [48]	Selective Laser Melting/SLM^2^ [49]; Selective Laser Sintering, SLS^2^ [50]; Direct Metal Laser Sintering, DMLS [51]; Electron Beam Additive Manufacturing, EBAM [52]	Selective Laser Sintering/SLS^2^ [32,53]
Sheet Lamination	Laminated Object Manufacture, LOM [54,55]	Laminated Object Manufacture, LOM [56,57]; Plate Diffusion Brazing/PDB [58]	Laminated Object Manufacture, LOM [32]
Vat photo-polymerization	Stereolithography/SLA [59]	Stereolithography [60,61]	Stereolithography/SLA [32,62,63,64]

^1^ This term is sometimes erroneously used as synonym for Additive Manufacturing in general; ^2^ SLM, SLS and DMLS very often describe almost identical processes.

The reader should note, though, that (a) this overview cannot cover the full scope of AM techniques and that, (b), different designations are around for almost identical processes, as many proprietary terms by equipment suppliers *etc.* have entered the literature. Our collection will give a first idea of the present diversity and direct the interested reader towards sources of further information.

As shown in Table 1, and based on the relevant ASTM terminology standard F2792-12a, the main processes currently in use for Additive Manufacturing can roughly be sorted in seven categories:

Binder jetting implies a bonding agent selectively deposited to join powder materials. Product geometry is determined, layer by layer, via this deposition step, while the matrix is provided as powder bed.

Directed energy deposition uses some deposition technique to furnish the matrix material and defines part shape in parallel. Building material typically comes in particulate form, and coherence is achieved by focused introduction of thermal energy via sources like laser, electron beam or plasma arcs.

In material extrusion, geometry is defined by matrix material feed via dispensing through a nozzle or orifice. Thus when being processed, the matrix has to be in fluidic form—a fully or partially molten polymer or metal, a slurry or suspension.

Material jetting resembles material extrusion, except for the fact that processes are discontinuous at the micro scale, as materials are provided as droplets, which implies higher fluidity/lower viscosity material formulations.

Powder bed fusion describes processes in which the building material is spread out as powder layer or bed, and in which energy is introduced locally to merge the particles e.g., by melting or sintering processes. Product geometry is defined by this fusion process, while material feed is geometrically undefined. This allows differentiation from directed energy deposition, in which materials are selectively deposited, thus creating the part geometry.

Sheet lamination is based on cutting the geometry required in a specific layer from a foil which is bonded to the preceding layer. Methods of bonding depend on the materials processed and include adhesive bonding applied to paper, polymer or metal foils as well as diffusion brazing of aluminum AlSi12 alloy-coated steel sheets for entirely metallic components (plate diffusion brazing/PDB, plate press brazing/PPB) [58].

Vat photo-polymerization has probably been, in the form of stereolithography, the one additive manufacturing process that first raised awareness of this class of processes among a wider public. Common to this and all neighboring processes is the use of photopolymers in liquid state which are selectively cured either via a mask—a less common variant, as it is less flexible-, or via localized energy input by means of UV laser scanning of the respective cross-sectional geometry [65].

**Figure 5:**
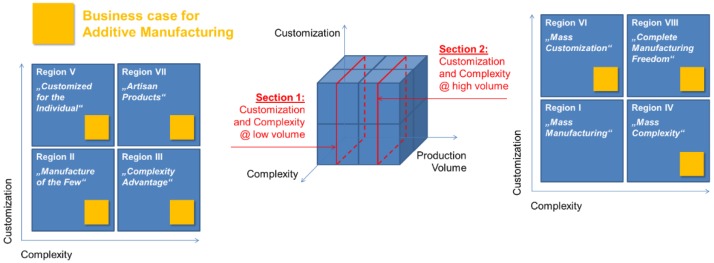
The defining regions of product characteristics in production volume-complexity-customization space and the respective potential of AM techniques according to Conner *et al.* [66].

In a landmark paper on AM economics, Conner *et al.* discuss the business case for AM techniques in different scenarios characterized by production volume, product complexity and degree of customization. The eight basic “regions” this model foresees are depicted in Figure 5 [66].

In their work, Conner *et al.* start from the notion that across industries, there are controversial opinions about the value and market potential of AM techniques. They take this controversy as a motivation for distinguishing between production scenarios (as exemplified in Figure 4) and discuss the relevance of AM based on this delimitation [66].

Region I is the classic realm of primarily cost-driven mass manufacturing. Investment in specialized manufacturing equipment can be extremely high in this scenario, as amortization is spread over very large series. Examples include e.g., sheet metal forming for automotive body structures. Directly, additive manufacturing typically has no footing here. Indirectly, it can contribute, though, not by providing the final components themselves, but the tools to make them: Of these, few are needed, and even using conventional processes like milling in the case of metal forming or casting dies, their production is a time-consuming affair. In contrast, rapid tooling via AM processes can offer superior speed and lead time reductions making it an example for Region II, “Manufacturing of the Few”. The classic idea of Rapid Prototyping falls into this category, too. Region III comprises products that profit from a level of complexity that cannot be achieved via conventional manufacturing processes at all, or at least not economically. Once again, examples may be found in the area of manufacturing tools, where conformal cooling may help to improve product quality and/or reduce cycle times in processes like plastic injection molding. The basic idea behind this application is to provide tools with cooling channels that ideally reflect the part geometry, e.g., by maintaining a constant distance to the inner mold surface. Subtractive manufacturing would require a modular built-up to come anywhere near to this, whereas AM allows geometry optimization based on the main functional aspects alone, *i.e.*, thermal, fluid flow and mechanical considerations. The main driver/enabler here is that, as Connor *et al.* state, “complexity comes at no cost” in AM [66]. Similar to the previous, but at higher production volumes, Region IV is characterized by low levels of customization, but again a high degree of complexity. AM processes can be competitive in this region if complexity provides enough added benefit to compensate lower cycle times achieved in conventional processes—or if the latter only reach a mandatory level of complexity in a sequence of processes that together is more cost- and/or time-intensive than the single AM process capable of replacing it. Region V adds customization as further parameter, with production volume and complexity assumed to be low. Personalized consumer products like mobile/cell phone casings with user-defined features fall into this category. On a more sophisticated level, customized implants, prostheses or orthoses might be envisaged, as long as complexity remains low. By stepping up in production volume, but maintaining limited complexity, Region VI is reached. The catchword here is “Mass Customization”, *i.e.*, the production of large amounts of individualized products. Thus a business case in this region requires a large market which profits from individualized solutions that would, realized via conventional processes. More complexity, but less production volume is what Region VII summarizes. Here, AM can become an enabling technology that provides the economic means to realize solutions that would otherwise be unaffordable. Again, medical technology comes to mind, and the possibility to adapt even complex prosthesis to the individual bearer. Finally, Region VIII describes, as Conner *et al.* put it, the “total manufacturing freedom”: High volume production is economic, and both complexity and customization carry no added cost. As Connor *et al.* state, this region has not yet been entered on a commercial basis—by way of an explanation, they suggest the still limited production rates and build envelopes of current AM systems. Besides, however, they point at missing links in the product development chain that impede the efficient take-up of e.g., personal medical data into prosthesis design [66].

Other motivations and business cases for AM in direct competition with conventional machining processes include the potential for reduced lead times [66] and time to market (TTM) in general, the latter with specific relevance for highly complex parts, where complexity is not only defined by geometrical or topological considerations, but also by integrated functionality—Macdonald *et al.* specifically stress the potential of processes that allow integration of electronic components in this respect [67]. Options of this kind will be treated in more detail below. Besides, repair has been identified as promising area of application for specific AM techniques, though the precondition usually is that processes rely on feed of material, which excludes approaches like powder-bed fusion, laminated object manufacture or vat photo polymerization in their classic form. From a more distant vantage point, replacement of faulty or damaged components is another option in maintenance and repair which moves the origin of spare parts into focus. Additive manufacturing promises nothing less than eliminating spare part logistics by producing any necessary component just where it is needed. Such a decentralized production scenario has been scrutinized by Khajavi *et al.* based on F-18 Super Hornet military aircraft component logistics, with the result that economically, current AM system performance levels and investment costs still favor centralized production. The foreseeable development of AM machines, including special designs adapted to the spare part task, however, are expected to reverse this result in a near future [68], with the maritime industry a potential new customer envisaging improved on-board repair capabilities [69].

**Figure 6:**
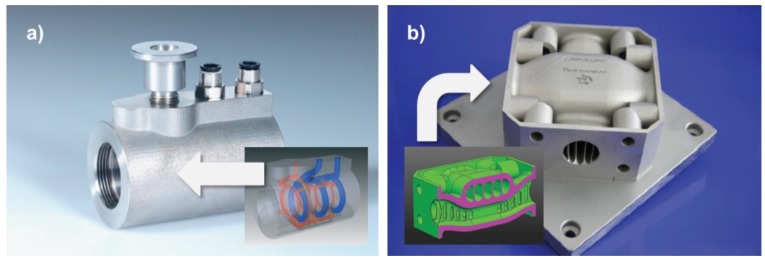
Examples of products and case studies representing major regions of interest of AM technology application: (**a**) A plastic injection molding die featuring conformal cooling channels [70], an example of the Rapid Tooling approach representative of region II and III according to Connor *et al.* [66] and as depicted in Figure 5; (**b**) low weight, low flow resistance hydraulic crossing [70], region III and IV.

Figure 6 provides two examples of AM products and application studies that reflect some of the major areas of interest as defined in the previous paragraph and figure.

#### 2.3.2. Approaches towards Sensor Integration in AM

Sensor and electronics integration to realize material-integrated intelligent systems, sensorial or robotic materials or structural electronics components is currently the object of many research activities fueled by application scenarios like structural health monitoring (SHM), smart skins for robotics and prosthetics and smart components forming part of the Internet of Things (IoT) [71,72,73,74,75]. In such contexts, interest in AM-based sensor integration is likewise on the rise. As a first step, such attempts require multi-material processing in AM, and in fact, such solutions are available. A fundamental example is processing of homogeneous composite or hybrid materials without gradients in composition, and without controlled orientations e.g., of reinforcing particles. Here, the main challenge is to rule out de-mixing of materials during the process. This is specifically critical when fractions of particulate materials deviate considerably e.g., in size or density. Tools for analyzing such problems include analysis of particle flow through discrete element method (DEM) modeling [76]. Despite this issue, several of the basic AM techniques can be modified for multi-material processing, e.g., by provision of suitable, stable powder mixtures, reinforcement phase particles coated with the foreseen matrix materials, and direct processing of particulate composite materials in powder bed fusion processes, by use of dispersions of a reinforcing material in a photopolymerizable material in vat photopolymerization or by adding the second phase as component of the binder in binder jetting processes. Kumar and Kruth, and more recently Vaezi *et al.*, have provided reviews covering the respective materials and process variants [77,78].

The challenge is much harder to tackle if a spatially defined change in composition or properties is envisaged, as this implies the need for locally controlled variation of materials and/or material properties. Guo and Leu thus distinguish between the aforementioned uniform composites on the one hand and functionally graded materials (FGM) on the other [79]: Compared to a layer-to-layer gradient along the building direction as most fundamental realization of an FGM through AM a new level of complexity is reached if variation needs to be clearly defined in three dimensions. This is the case when sensors, electric and electronic interconnects and finally electronic components themselves are to be generated during the building process, using the same manufacturing system. The promise of such components, and the potential of AM to deliver them, has long since been recognized [80]: It is a fundamental aspect of our current scenario.

Suitability of AM process classes for the task varies depending on the principle of shape generation: Processes which establish object geometry by locally consolidating a geometrically simple volume of material are less suited than those in the course of which the material is deposited locally. The latter facilitate a change of material during the building process, e.g., by switching between reservoirs in dispensing or extrusion processes. Material jetting and material extrusion, but also directed energy deposition processes fall into this category—powder bed fusion and vat photopolymerization represent the opposite class. Binder jetting, in contrast, assumes an intermediate position, as this process combines local deposition of material with a material reservoir, notably a powder bed. Here, the possibility of applying the binder to locally modify the powder bed material has been demonstrated with respect to spatial control of carbon content levels, and thus of hardenability, in steel parts [81].

Present attempts at sensor integration typically follow either of two different paths: The first is best described as combination of production processes, where separate processes like functional printing ([82,83]) account for adding secondary and ternary materials serving functional purposes like sensing or electric insulation (see Table 2, manufacturing principle “separate process”, subclasses “part/part section as substrate” and “separate process, hybrid manufacturing system”). The second option is ex-situ production of sensing elements and integration of these as subcomponents in the course of the build-up of the final part (“separate process & substrate” in Table 2). Essentially, it combines assembly via pick-and-place operations with the classic, additive building processes. The former strategy is attractive because of the neat integration of sensors into the structure. Depending on the underlying AM process, it can either be realized by using multiple manufacturing systems intermittently (ex situ), or by implementing secondary processes suitable for sensor integration in an AM manufacturing system—typically this could be a printing process (in situ). The second approach, on the other hand, allows for integration of intelligent sensor systems rather than individual sensors and is feasible already today.

The obvious third option, and a long-term objective at that, would need adaption of the AM process itself towards an in-process switch of materials, including the option to create geometrically defined structures from all materials involved (“same process” in Table 2). For component-integrated sensors, this approach will require handling several materials in parallel, since conductive paths and insulations thereof as well as the different integrated components’ functional requirements would demand highly specialized individual materials. Due to this complexity, for the time being multiple processes in a single setup seems a viable intermediate step.Besides, since achievable levels of complexity even in upgraded or hybrid AM systems will fall short of dedicated IC production process capabilities for any foreseeable future, combination of direct building and assembly techniques will remain the preferred short- and mid-term path towards highly capable sensorized AM parts.

**Table 2:** Exemplary case studies on AM products with integrated or applied sensors, sensors systems, PEIDs *etc.*

Integration Level	Manufacturing Principle	Description (Principle)	Material (Product)			AM Process	Reference
			Polymer	Metal	Ceramic		
1—surface application & integration	1.1—separate process & substrate	1.1.1—Application of SMT components on the surface of AM parts.	X			SLA	[84]
	1.2—separate process, part as substrate	1.2.1—Aerosol Jet printing of conductive paths for powering LEDs and propellers of a small scale UAV produced via FDM process.	X			FDM	[85]
		1.2.2—Provision of interconnects for surface-mounted devices via Aerosoal Jet printing on curved surfaces, linked to example 1.1.1.	X			SLA	[84]
2—2D volume integration	2.1—separate process & substrate	2.1.1—Adaptation of 3DP process for creation of cavities to hold electronic components, integration and electrical connection of same using inkjet printing		(X)	(X)	3DP	[84]
	2.2—separate process, part (section) as substrate	2.2.1—Ultrasonic Cu wire bonding on FDM substrate and subsequent over molding as encapsulation step for production of interconnects between electronic components and of sensors.	X			FDM	[86]
		2.2.2—Screen printing of insulating layers on Al foil, joining of foils using a sonotrode in an ultrasonic additive manufacturing (UAM) variant of the LOM process		X		UAM/UC	[87,88]
3—3D volume integration	3.1—separate process & substrate	3.1.1—Manual placement of separately manufactured electronic components (LEDs) and/or sensors within cavities of a SLA part.	X			SLA	[67]
	3.2—separate process, part as substrate	3.2.1—3D and cross-plane via holes for electrical interconnects produced in a 3DP ceramic part, conductivity achieved via electro-less copper plating adapted from PCB production, surface mounted electronic components according to a 3.1 principle.			X	3DP	[89]
	3.3—separate process, hybrid manufacturing system	3.3.1—SLA system upgraded with an ink dispenser system for direct writing of laser-cured silver ink based conductive paths, suitable for 2D and 3D paths.	X			SLA	[88]
		3.3.2—FDM combined with inkjet printing system in a single, commercially available device offered by startup company Voxel8. Full 3D-integration of conductive paths *etc.* plus external, manual pick-and-place capability.	X			FDM	[90,91]
**Integration Level**	**Manufacturing Principle**	**Description (Principle)**	**Material (Product)**			**AM Process**	**Reference**
			**Polymer**	**Metal**	**Ceramic**		
3—3D volume integration	3.3—separate process, hybrid manufacturing system	3.3.1—SLA system upgraded with an ink dispenser system for direct writing of laser-cured silver ink based conductive paths, suitable for 2D and 3D paths.	X			SLA	[88]
		3.3.2—FDM combined with inkjet printing system in a single, commercially available device offered by startup company Voxel8. Full 3D-integration of conductive paths *etc.* plus external, manual pick-and-place capability.	X			FDM	[90,91]
		3.3.3—Hybrid manufacturing system comprising FDM, ink dispensing, thermal embedding of wires, micromachining, robotic pick & place assembly in one system to allow “single setup” realization of smart objects.	X			FDM	[92]
		3.3.4—Placement of separately manufactured SMT components within cavities of a 3D Printing part created via integrated suction-based powder withdrawal system, also used for automated pick-and-place operation.		(X)	(X)	3DP	[84]

**Figure 7:**
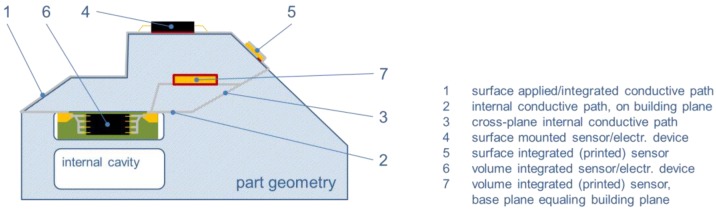
Overview of possibilities for combining sensors and sensor or smart systems with AM parts, inspired by a similar schematic image by Hoerber *et al.* [84]. The numbering corresponds to categories in Table 2 as follows: No. 1 matches category 1.2, 2 equals 2.2, 3 = 3.1 & 3.3, 4 = 1.1, 5 = 1.2, 6 = 3.1 & 3.3, 7 = 3.3.

Figure 7 summarizes the possible variants of sensor and sensor or smart system integration in an AM part based on a similar depiction by Hoerber *et al.* [84]. Table 2 grants an overview of studies detailing several of these and sorts them according to the achieved level of integration and the manufacturing principle applied. The former extends from external (surface) application via surface integration to volume integration. As discussed, volume integration can once more take two forms, the first comprising approaches in which the individually built-up production layers act as defining planes for an essentially two-dimensional integration, the second a true three-dimensional integration which transgresses these limits. In the following, we will speak of 2D *vs.* 3D volume integration wherever it is necessary to highlight the difference. Beyond integration level, the manufacturing principle as developed in the preceding paragraph helps structuring the table.

Macdonald *et al.* have recently provided a study, also cited in Table 2 above, in which 3D volume integration of conductive paths is combined with individual components the addition of which constitutes the full circuit. The product is a gaming die capable of detecting its position and indicating it via integrated LEDs that represent the typical face patterns associated with the various numbers. The basic process used is stereolithography, during which buried tracks for conductive paths and openings for electronic components are foreseen in the AM part, with component placement realized through state of the art pick-and-place operations and filling of the tracks with printable inks, both necessarily done separated from the preceding AM step. As the process chain relies on 2D design of the electronic system, and folding of the respective planar layout to achieve positioning on the six faces of the die, in Table 2, the study must be considered an example of 2D surface integration in a separate process and using the final part as substrate. As a specific challenge, Macdonald *et al.* mention the lack of software tools for true 3D layout of circuitry, as the available systems typically address planar PCB design and allow at best folding of the finished 2D design, or wrapping it around geometrically simple objects. In effect, the full potential of 3D integrated electronics cannot be realized, and for the present system, MacDonald *et al.* had to rely on a combination of conventional circuitry layout and 3D CAD systems [67].

In a follow-up study by the same working group, Espalin *et al.* describe the features of an integrated manufacturing system combining several production and assembly processes to overcome some of the shortcomings of the previous device. Among these are the strength of SLA matrix materials and the conductivity of interconnects realized via dispensing of inks, which were both felt to be too low. The solutions adopted by Espalin *et al.* include switching from SLA to FDM and thermoplastics as well as adding a system for copper wire thermal embedding [92]. About the latter, Shemelya *et al.* report in more detail [86]. The ink dispenser system, however, was retained for realizing those interconnects either not requiring highest conductivity levels, or transgressing the building planes. Since FDM resolution does not attain the respective SLA capabilities, for building channels to hold ink-based conductive paths at sufficiently low pitch values, a micromachining system was added. Besides, an automated pick-and-place system is available for integration of electronic components in the final part. Not yet a part of the full, multi-technology manufacturing system, but externally tested and suggested for integration is a laser micro welding system capable of joining electronic components and conductive paths. Similarly, laser ablation is considered as alternative to micromachining for realization of cavities and high-resolution channels for conductive paths. Currently the resulting multi^3D^ system is probably the most sophisticated solution for realizing electronics-embedded polymer components. Its fundamental philosophy is integrative, and not generic, though [92]. A somewhat simpler, but basically similar system is offered on a commercial basis by the Arvard University spin-off Voxel8, which offers a printing system that comprises a filament-based FDM process in combination with an integrated inkjet printhead. On top of this dual-process setup, building platform positioning and fixing has been adapted to allow interruption of the building process, removal of the building platform for external processing (e.g., pick-and-place operations for addition of electronic components), replacement of the building platform and continuation of component build-up right from the point where it was interrupted [90].

Summing up the current state of research on embedding sensor systems in additively manufactured structures, to the authors’ best knowledge, the following deficiencies prevail:

There is no single process that allows, through variation of materials and/or process parameters, the direct realization of 3D volume integrated sensor systems, or has been evaluated towards this end.

Polymer-based solutions dominate the field, since both metals and ceramics typically mean harsher manufacturing conditions, and metals increase complexity further by requiring insulation layers separating functional units from the (conductive) bulk.

Technologies like printing of conductive paths often fail to reach theoretically possible performance levels because of limitations imposed by other system components, e.g., thermal stability of polymers capping the applicable sintering temperatures.

Of all available options, directed energy deposition, with the LENS^TM^ [33], DLF [34] and DMD [35] processes as examples, is probably the most likely candidate for overcoming all these obstacles in a single process, as the principle allows switching of materials easily and is suitable for handling metals and ceramics. If sensor integrated AM parts in general are sought for, however, a suitable combination of processes may seem a more favorable solution. Conclusion is, however, that in commercialization of such integrative solutions, it is not so much new technological breakthroughs that are required, but a further development and optimization of existing concepts to support economic viability. Voxel8 has done a very important first step in this direction [90]. Thus for the realization of the present paper’s vision, the ground is already prepared.

### 2.4. Usage Data Supported (Automated) Design

In this subsection, various state-of-the-art design approaches using usage or PLM data, collected by sensors or otherwise, are elaborated. Furthermore, a snapshot of different application cases and current research projects related to this topic is presented. Automation of design tasks is subject of research for at least two decades. In this paper, two main approaches for design automation are described, *i.e.*, Knowledge-Based Engineering (KBE) and the design of adaptive products.

As design is a knowledge intensive task, the research stream of KBE investigates approaches to capture and re-use product and process engineering knowledge. KBE seeks to automate repetitive and non-creative tasks, however, leaving more difficult decisions to (human) designers. The principles behind the automation of repetitive design tasks is often facilitated by the use of Ontologies, semantic connection of knowledge and a framework of independent formalization of knowledge [93]. Criticism towards current KBE approaches include their mostly case-based nature, black-box application, lack of knowledge re-use, lack of quantitative assessment of cost and benefits and thus the lack of a quantitative framework to judge the value-add of KBE development [13]. On the other hand there are several successful examples of KBE implementation available. If successful, this offers not only more time for human designers to focus on advanced design tasks.

Another approach of design automation concerns the integration of evolutionary concepts, lent from biology, into engineering design. Beal suggests to apply self-organization approaches in engineering to enable adaptive design [14]. Products having the ability to adapt, are more robust in situations where initially defined system requirements change. According to Beal, a key concern, however, is that initial product design becomes more difficult, as adaptation problems must be faced that would be otherwise postponed or overlooked. A similar attempt to combine engineering and evolutionary concepts is summarized by Denkena *et al.* [94]. Their research work was conducted over several years within the Collaborative Research Center 653 “Gentelligent Components in Their Lifecycle” (Germany). The approach aims for the collection and storage of product information from within the product with minimum additional electronic components. The scenarios include modification of materials to make them sensitive, in a very broad sense, e.g., to mechanical loads experienced in use. An example is the mechanically induced martensitic transformation of non-magnetic austenite phases in steel. The ensuing change in magnetic characteristics can be detected upon inspection by means of an external sensor system, or continuously via surface-attached or surface-integrated sensors. Its level is interpreted as an indication of peak loads experienced by the component. In an associated case study, these data are used for mechanical structure optimization of a wheel suspension by employing a genetic algorithm and a parametric design model [95]. Parametric design models allow the rapid adaption of design features by incorporating design rules into a design model. These rules take into account that the change of one parameter of a product (e.g., a length of a handle) may impact other features of the product. Hehenberger states that products have different degrees of standardization (or likewise parametrization) depending on cost and quality aspects [96].

In terms of design automation, there are also associated approaches that do not seek to automate the creation of actual designs, for instance in the form of CAD models, but instead focus on making the identification of product failures easier. Product failures affect user satisfaction and thus must be addressed by producers. The established “rule of ten” states that failures, identified early in the process are by magnitudes less costly than failures identified during the usage phase [97]. If failures can be traced back to design shortcomings, the design can be adapted. Example cases that apply usage information, in order to detect and address product failures are provided in [11,98,99,100]. A selection of use cases applying usage information in the context of product design are summarized in Table 3.

**Table 3:** Examples of products collecting usage information to support design decisions.

No.	Product/Area	Ref.	Purpose/Goal	Considered Usage Information (Examples)
1	Hydraulic system	[101]	Identification of weak points of parent generation and support in design of next generation	Temperature (inside system and environment), pressure, rotation speed, vibration, time of use, load during use, time of failure, kind of failure
2	Rotary spindle unit	[100]	Fault diagnosis of drive belt using Bayesian network	Spindle rotation speed, spindle running time, ambient temperature, last maintenance
3	HVAC	[102]	Identify potential product failures	Not explicitly mentioned.
4	Robot	[14]	Design adaptation using self-organization	Not explicitly mentioned.
5	Furniture	[103]	Quantification of furniture deterioration using a sensor network	Human use of the product and product wear, pollution, light, relative humidity
6	Vehicle fuel injection system	[104]	Method for use of warranty data to improve products	Warranty data (not further specified)
7	Leisure boats	[20]	Improvement of boats in general	Atmospheric pressure, external temperature, air humidity and engine RPM, geo position
8	Spur gear manufacturing	[105]	Improvement of manufacturing process	Geometry information of the currently produced gear
9	Injection molding machine	[106]	Support for finding temperature inconsistencies during molding process	Temperatures, pressure of the turning screw, produced product quality (binary)
10	Machine tool (spindle)	[107]	Remaining useful component lifetime estimation	Vibration, motor power, rounds per minute and speed, temperatures, number of start/stop and acceleration/retardation, travel length, number of alarms, running time
11	Wheel carrier component	[95]	Identification of develop-relevant load cases and adaptation of design using a genetic algorithm	Forces
12	Boats	[108]	Estimate potential for improvement of boats	Service information, boat data, customer-provided data (not further specified)
13	Locomotive	[11]	Correlation of design parameters with field data that influence working status of product	Brake cylinder pressure, locomotive acceleration, heating circuit current, filter current, catenary voltage, mileage

Most of the cases described in literature focus on the identification of failures and their root causes. The actual automated change of design parameters, however, is rarely addressed. The examples indicate that a variety of actual information is collected and used. Besides information about the environment (e.g., temperature and humidity), also performance indicators of the product are relevant (e.g., acceleration and power consumption). In case of production machinery (or products creating a usable output), also quality parameters of the output are relevant (e.g., geometry).

## 3. Usage-Data Enhanced (UDE-) CBDM: A New Process Model Rooted in Cloud-Based Services

The underlying concepts of CBM, CMfg and CBDM have been discussed in the introductory section. The proposed criteria, describing the extensions we recommend, have also been formulated in that section (Figure 2). Based on these added requirements, the state of the art in the respective technological domains has been scrutinized in Section 2: apparently, most of the basic ingredients needed for an extension of CBDM towards UDE-CBDM are already available or at the very least under development. Despite this encouraging fact, we hope to be able to pinpoint a number of white spots that still deserve further research efforts, and more that need development towards commercialization: These will in part be introduced here, illustrated by the case studies, and detailed in the discussion.

**Figure 8:**
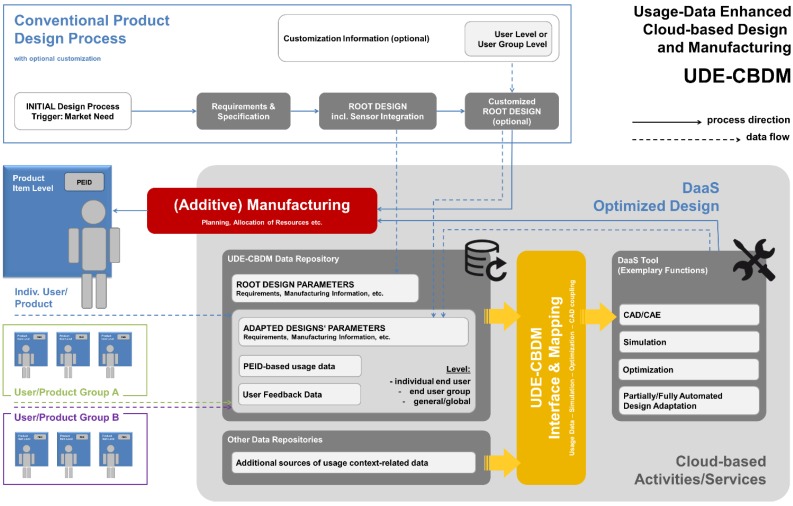
Process scheme describing main activities and data flows in usage-data enhanced cloud based design and manufacturing (UDE-CBDM).

Figure 8 outlines the process sequence we proposed in terms of a process direction and data flows linked to the individual process steps.

We assume that the initial motivation to introduce a new product, as well as its initial design, both follow conventional approaches: We call this initial design the root design, as it is the common ancestor and thus the foundation of all variants that may be generated at later stages within the scheme. This root design may, in a subsequent, optional step, be customized to meet the requirements of a specific individual user, or a user group. Descriptions of both designs will then, as the vertical data flow paths suggest, be added, as initial entries, to a related data repository: In it, the root design description is of global character, whereas a possible adaptation to a user or user group would bear some reference to these. The provision of the respective data repository denotes the first cloud-based service encountered in the processing sequence.

In the case of the initial product variant, the next steps would focus on tasks directly associated to the manufacturing processes. This includes the identification of suitable manufacturing resources and the allocation of the manufacturing task to these, based e.g., on a cloud-based quoting system as suggested in several CBDM definitions. An added feature in a real-world implementation of UDE-CBDM is that manufacturing would heavily rely on AM processes. These would contribute their generic flexibility, thus facilitating almost immediate fabrication of product variants representing new adaptations of the root design. The “almost” in the preceding sentence is owing to the fact that new geometries will require a revision of the manufacturing, *i.e.*, build-up, strategies (e.g., scanning patterns in SLS, SLM or SLA processes), in order to minimize adverse effects like residual stresses and the like: The assumption is that initial information on this issue may have been provided with the root design, but any geometry or material-related alteration would require further checking, and almost invariably updating of the respective information for the adapted design. The general UDE-CBDM concept leaves open whether such tasks are associated with the DaaS toolset, or alternatively with the manufacturing side. Handling this at the design stage would be the preferred option, though, to make sure that no design unfit for production is issued. Any design adapted by the DaaS tool would be added to the UDE-CBDM data repository. Assuming a highly parametric design, this could be done, for instance, through storage of the relevant design parameter values—or at least of those that deviate from the root design. The adapted design should then also be linked to the data set on which it had been based. The reasoning behind this measure is that a new design for the same user or user group could potentially be derived more easily from an evolved product state than from the initial, root design.

Collection of usage data would start at the latest following the delivery of the product to the customer. This data would again find entry into the aforementioned data repository, where it would be associated with the root design and any pre-existing design variant, as well as to the individual user and/or to the user group he/she belongs to.

Any triggering event that would initiate production of a second variant of the root design would not be channeled into the conventional design path, but would rather be diverted to the DaaS tool offered as a cloud-based service. This tool would draw on root design information and usage information available from the UDE-CBDM data repository. Besides, direct user feedback and information might be available from the latter. Finally, if connections between product usage data and other cloud-based sources of information can be made, additional information, e.g., on the usage context, could be gathered. Interfacing and mapping between the data repository and DaaS tool would require a dedicated tool, which would constitute a central element of the entire concept.

Depending on the capabilities of the DaaS toolset, the concept could allow the customer to directly trigger the manufacturing process simply by placing his order, up to a level without any human intervention in a fully automated design process linked to a likewise automated process handling the manufacturing organization up to the final logistics that would guarantee that the product reaches the user. From a business model perspective, this notion has several interesting implications—companies that originally developed the root design might not be selling products then, but licensing designs instead. Alternative DaaS provides might compete for customers. Access to usage and associated data might become a trading good and as such be offered by specialized service providers. We will discuss such issues in a little more detail in Section 5, and specifically in Section 5.1 and Section 5.2.

With more and more products sharing a common root design on the market, the UDE-CBDM data repository would grow, allowing more and more discrimination between user groups *etc.*

The separation between the establishment of a root design on the one hand and an initial customization on the other makes sense because, in order to facilitate the later design adaptation, definition of this root design must be flexible in any case. Enhanced parametric design could be a useful approach in this respect, as it would provide the main design parameters as initial, primary handles for adaptation. Foreseeing this facility from the start could then also support any initial customization, if needed. In an implementation of this kind, however, the initial designer must have in mind that the way in which his root design is parameterized may determine the envelope within which future design adaptations can occur, as any deviation from this framework might require a partial re-parameterization of the design, and thus a considerable extension of both width and depth of the design adaptation task. In practice, this would imply that automated design adaptation might only reach local, not a global optimum, depending on the level and extent of any pre-determination contained in the fabric of the root design.

The DaaS toolset itself can be seen as a very specific and very complex example of (at least) a SaaS system. Due to the complexity, which will be discussed in Section 5, where we intend to show that not only the designer perspective, but also the regulatory one is of primary importance with respect to this service, we have nevertheless opted to coin the DaaS label here.

## 4. Case Studies

In this section, three case studies transfer the previously presented UDE-CBDM concept to actual application environments. The aim of this section is not to present a comprehensive and evaluated “ready-to-use” solution, but to show the potential of the vision and provide a basis for future critical discussion of the topic. The first case study focuses on medical implants, which are by definition one-of-a-kind and highly customized based on the users’ preferences, state and physique. At the same time, they are not extremely complex compared to other multi-component products. Hence, this environment provides a solid frame for the envisioned application, and a frame at that in which Additive Manufacturing is already a competitive production process and sensor integration has been used to derive valuable usage information on a general, not individual level in the course of several research projects. The second case study describes “the other extreme”, with a scenario set in the robotics domain. In this case, the natural ability of robots to collect and communicate usage data through their own, not necessarily monitoring-, but task-oriented sensors and communication equipment is used in order to enhance this implementation. The focus is very much on data processing techniques, with agent-based solutions identified as the method of choice, and the linking of these to the overall scenario as laid out.in Section 3.The section is closed by a third case study representing a more detailed study of a scenario based on the design and production of shoes which foresees usage data backflow and personalized design solutions based on these. This example is based on a dedicated research project conducted in Japan, which as such goes beyond the preceding studies in being much nearer to implementation. Not covered at present is the envisaged automated redesign capability provided by the DaaS toolset.

### 4.1. Case Study 1: Medical Implants—Generation-to-Generation Adaption Based on Strictly Individual Use Patterns

For several types of medical implants, consideration and even practical application of AM processes is commonplace, as by definition, they need to be individual in their adaption to an individual patient [109,110,111]. Business cases in this respect have been highlighted in Section 2.

A general aspect of several types of implants is that they are subject to influences like wear and degradation and thus may need replacement. Any such replacement means surgery, with all the risks this entails for the patient. This constitutes a strong motivation for increasing the lifespan of an implant. Hip implants, to consider a concrete example, are both common (at 300,000 replacements performed in the United States in 2010 alone and 80,000 annually in the UK, according to Kandala *et al.* [112]) and heavily loaded. At the same time, they are subject to revision surgery in a relevant number of cases. Studies from the UK have considered revision rates after a given time as a benchmark for different products and indicate that an aim should be set to achieve, for a given product, a likelihood of revision of less than 5% within a 10 year period [112].

If production of the implant in question is AM based anyway, manufacturing of an altered part is a realistic option, if there is a sound justification of a redesign, and if economics of the redesign process can be covered: manufacturing costs, in contrast, are unlikely to change significantly compared to the initial part. However, the primary concern when it comes to redesigning an implant for the individual patient is the availability of personal data. The High Road to gathering such data is equipment of the implant with a suitable set of sensors allowing read-out of raw data or processed information at any time. Smart implants of this kind have been realized for several body areas, including the aforementioned case of a hip replacement, but also the shoulder, spine or knee, as bone or cartilage substitutions—an overview in this respect has recently been published by Ledet *et al.* [113]. Sensor-integrated systems of this kind have provided extremely valuable information for guiding implant design in general terms by measuring forces, moments or temperatures associated with different designs and different use patterns. They have furthermore contributed to an improved understanding of the musculoskeletal system, and of healing processes following the implantation—all this on a level that would hardly have been accessible through other means. However, in performing these tasks, up to the present day, they have remained tools for research rather than becoming a standard aid to the implant designer, far less the average patient.

Going back to the example of the hip implant, typical early smart implant designs incorporated three-axis force measurements [114], later ones recorded bending moments and temperature, too [115]. In the latter variant, sensor system and signal processing are integrated in the hip implant’s neck, while the antenna used for telemetric data transmission to an external evaluation unit is located inside its ceramic head. The implanted system receives the necessary power via inductive coupling using a coil linked to the external evaluation and power supply unit, and a matching one again integrated with the implant [115,116,117]. System sizes have dropped from cm to the mm length scale reached today.

Besides smartness, adaptation of implants to the specific requirements of individual patients is a constantly studied issue, but the point of attack is typically not information on the patient-specific usage: In a recent study, Campoli *et al.* report about reconstruction of the link between internal bone structure and musculoskeletal forces using CT data, FEM modelling and optimization techniques for a scapula [118,119]. Similar principles could be used to determine likely implant loads and in this role might at least partly replace direct measurement of forces through smart implants, or compensate their non-availability when first implanting the respective bone or joint replacement. However, approaches of this kind, though building on observable individual structures and geometries, have to rely on load cases that are mere conjectures. Besides, they are extremely complex and incorporate several assumptions e.g., in terms of *in vivo* mechanical characteristics of bones and tissue, which makes automation a major area of study in this field [120]. In effect, this means that nowadays patient-oriented implant adaptation primarily addresses the geometry of interacting surfaces, as in the case of the hip joint, or the geometry of the bone in which the implant would be embedded, *i.e.*, the femur in the case of a hip replacement. Consideration of local density variation of the femur may similarly be taken into account based on CT measurements and associated local density derivation [121].

**Figure 9:**
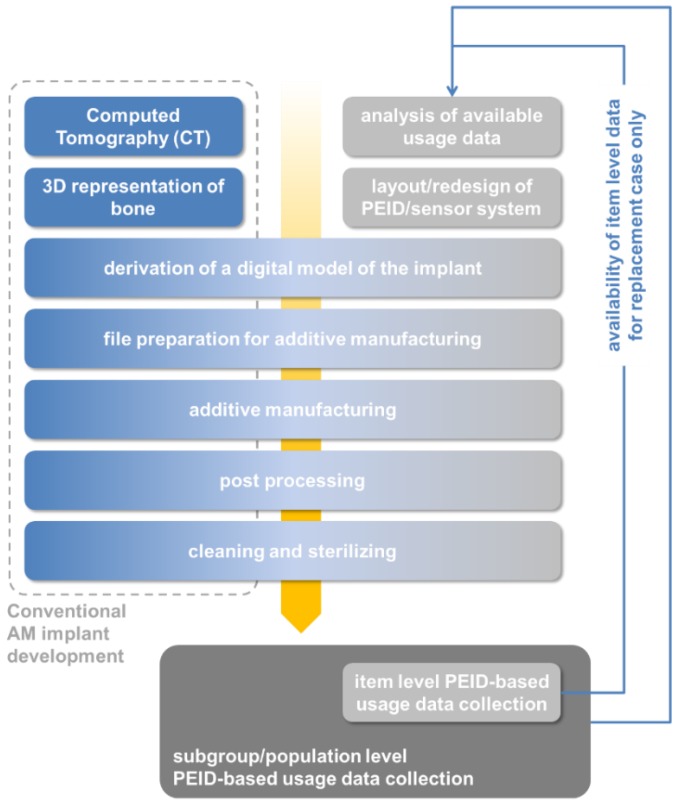
Typical design flow for implant layout according to Cronskär *et al.* [121] with additions introduced by a PLM-data supported approach combining item and subgroup/population level information. The diagram assumes PEID integration as integral part of the AM process.

Additive manufacturing of various types of implants has gained in importance in parallel to the rise to maturity and fame of the technology and as a means towards realizing the aforementioned patient-specific adaptations. A recent study by Cronskär *et al.* has demonstrated a cost advantage of 35% when manufacturing a series of 7 hip stem implants using EBM-AM rather than conventional (subtractive) manufacturing techniques [121]. The basis for implant layout, irrespective of the production path chosen, is a CT scan and a derived 3D representation of the receiving bone (femur). This established design approach is illustrated in Figure 9 and contrasted to its UDE-CBDM counterpart.

What is currently still missing is the suggested combination of all three of the above elements, *i.e.*, the integration of sensors and sensor systems, the optimization of structural build-up, and the realization via AM processes. In an implementation of the scenario proposed in the present study, the initial design of an individual hip implant could be optimized according to information from a general pool of data on hip implant use patterns. For a specific patient, information from the respective database could be filtered based on the patient’s physical attributes and general knowledge about use patterns. In the latter respect, personal data from other sources, such as body area networks or smart watches, could also be drawn upon. Data sources would thus be both the UDE-CBDM and the “other” data repositories included in Figure 8. The implant itself would be produced by means of a suitable AM process including the integration of the required set of sensors, plus peripheral electronics, energy supply systems (e.g., harvesting and storage-based) and wireless communication hardware. Here, the remaining limitations of AM processes in terms of sensor integration come into play. Information extraction could be permanent and patient centered, or performed during checkups. In case of a pending replacement, the next generation implant could either be designed in direct accordance with what the collected data suggests, or using predictions that derive trends from the individual’s data. These data would originate from a larger database, from which, depending on the specific case, comparable and matching data (e.g., age, gender and use pattern) could be derived. The new implant would once again contain sensor systems for a further iteration. The aim, however, would be to increase the timespan between replacements with this new design.

What makes this scenario a relatively simple one is the fact that the boundary conditions are mostly static here, *i.e.*, it is unlikely that the collected user data will suggest switching the principal load cases that governs dimensioning. Instead, the accumulated data will only modify the loads themselves, and thus change the design criteria merely on a parameter level, rather than on any disruptive one. Reversing this argument, we have to conclude that optimization must be realized on a very fundamental level in this case, like local variation of material properties, which can be achieved via control of porosity levels. Such designed local porosity levels have already been the object of intense study in the context of implants, and tools exist that are capable of optimizing them in relation to the relevant load cases and with AM the method of choice for production [122].

For the maker of the implant, the implied business model is attractive, too, because the need to retrieve and evaluate data from the previous device can be exploited to establish a customer tie, to a mutual benefit: What is sold with the first implant is not only a physical product, but a product with associated services, which open up an additional source of revenue for the vendor, and ensure optimum product performance for the bearer. What the scenario still lacks in practice is the technological capability of extending the data collection to any implant produced, and to do so in an AM approach. Besides, the automated DaaS toolset and the connection between it and the collected usage data plus associated information is not available yet. Well-matched optimization techniques, in contrast, have already been demonstrated as stand alone solution.

### 4.2. Case Study 2: Robots as Intelligent Products—Agent-Based Cloud-Manufacturing Supported by Product-Inherent Perception

This second case study outlines approaches for unified information processing architectures that are suitable for additive and adaptive manufacturing based on a closed-loop sensor processing approach, *i.e.*, PLM by back propagation of inherent product perception. This approach combines distributed data mining concepts (big data), sensor data processing, and the cloud computing (Internet-of-Things) paradigm. Additive and adaptive cloud-based design and manufacturing are attractive for the field of robotics, targeting both industrial and service robots as well as semi-autonomous carrier robots. In cloud-based manufacturing, the user of the product is integrated in the distributed design and production process [9] through cloud-integrated computing and storage solutions.

**Figure 10:**
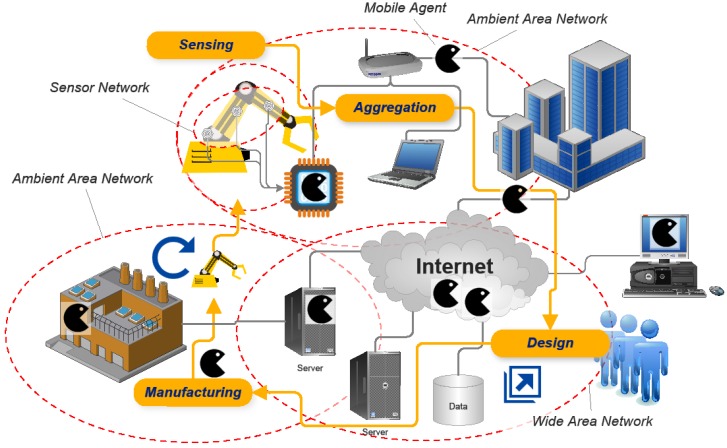
Additive and adaptive Manufacturing with back propagation of sensing data using mobile agents from robots to the design and iteration process resulting in continuous series improvements.

Robots can be considered as active, mobile, and autonomous data processing units, *i.e.*, hardware agents that are commonly already connected to computer networks and information processing infrastructures. Robots use inherent sensing capabilities for their control and task satisfaction, commonly delivered by integrated sensing networks with sensor preprocessing. These derive some inner state of the robot, for example, mechanical loads applied to structures of the robot or operational parameters like motor power and temperature. The availability of the inner perceptive information of robots enables the estimation of working and health conditions initially not fully considered at design time. The next layer in the cloud-based adaptive manufacturing process can be the inclusion of the products themselves delivering operational feedback to the current design and manufacturing process, leading to a closed-loop evolving design and manufacturing process with an evolutionary touch, shown in Figure 10 below. This evolutionary process adapts the product design, for example the mechanical construction, for future product manufacturing processes based on a back propagation of the perception information (*i.e.*, recorded load histories, working and health conditions of the product) collected by living systems at run-time. The currently deployed and running series of the product enhances future series, but not in the traditional coarse-grained discrete series iteration. This process can be considered as a continuously evolving improvement of the robot by refining and adapting design parameters and constraints that are immediately migrated to the manufacturing process. A robot consists of a broad range of parts, most of which are critical for system reliability. The most prominent failures are related to mechanical and electromechanical components, and are typically caused by overload conditions at run-time under real conditions not considered or unknown at initial design time. Furthermore, monitoring and profiling of the embedded software can be used for improvement and revision processes in the software design, which can be correlated with other perceptive data.

The integration of robots as products and their condition monitoring in a closed-loop design and manufacturing process is a challenge and introduces new distributed computing and data distribution architectures in strong heterogeneous processing and network environments. One major question to be answered is the sensing of meaningful condensed product condition information and the delivery to the designer and factory. It is a similar issue that arises in the Internet-of-Things domain. New unified data processing and communication methodologies are required to overcome different computer architecture and network barriers. One common distributed and unified data processing model is the mobile agent, a self-contained and autonomous virtual processing unit. The mobile agent represents a mobile computational process that can migrate in the Internet domain and in sensor networks as well. Multi-agent systems (MAS) represent self-organizing societies consisting of individuals following local and global tasks and goals. A MAS is a collection of autonomous or semi-autonomous problem solvers [123], often working proactively. This includes, for example, the coordination of information exchange in the design and manufacturing process [124]. The agent’s action and behavior depends on an abstract model of the environment described by ontologies. A MAS relies on communication, negotiation, collaboration, and delegation with specialization including instantiation of new agents.

Agents are already deployed successfully for scheduling tasks in production and manufacturing processes [125], and newer trends enquire into the suitability of distributed agent-based systems for the control of manufacturing processes in a wider sense [126], facing not only manufacturing itself, but maintenance, evolvable assembly systems, quality control, and energy management aspects, finally introducing the paradigm of industrial agents meeting the requirements of modern industrial applications. The MAS paradigm offers a unified data processing and communication model suitable to be employed in the design, the manufacturing, logistics, and the products themselves.

The scalability of complex industrial applications using such large-scale cloud-based and wide-area distributed networks deals with systems deploying thousands and up to a million agents. But the majority of current laboratory prototypes of MAS deal with less than 1000 agents [126]. Currently, many traditional processing platforms cannot yet handle big numbers with the robustness and efficiency required by industry [127,128]. In the past decade the capabilities and the scalability of agent-based systems have increased substantially, especially addressing efficient processing of mobile agents.

There are programmable agent processing platforms that can be deployed in strong heterogeneous network environments [129], ranging from single microchip up to WEB JavaScript implementations, all being fully compatible on operational and interface level, and hence agents can migrate between these different platforms. A distributed coordination and management layer enables the composition of large-scale agent processing networks [130] including WEB browsers creating one big virtual machine. Multi-agent systems can be successfully deployed in sensing applications, for example, structural load and health monitoring, with a partition in off- and online computations [131]. Distributed data mining and Map-Reduce algorithms are well suited for self-organizing MAS. Cloud-based computing, as a base for cloud-based manufacturing, means the virtualization of resources, *i.e.*, storage, processing platforms, sensing data or generic information.

Traditional closed-loop processes request data from sources (products, robots) by using continuous request-reply message streams. This approach leads to a significantly large amount of data and communication activity in large-scale networks. Event-based sensor data and information distribution from the sources of sensing events, triggered by the data sources (the robots) themselves, can improve the allocation of computational, storage, and communication resources significantly and limit the need for these.

A cloud in terms of data processing and computation is characterized by and composed of

- a parallel and distributed system architecture,
- a collection of interconnected virtualized computing entities that are dynamically provisioned,
- a unified computing environment and unified computing resources based on a service-level architecture, and
- a dynamic reconfiguration capability of the virtualized resources(computing, storage, connectivity and networks)

Cloud-based design and manufacturing is composed of knowledge management, collaborative design, and distributed manufacturing. Adaptive design and manufacturing enhanced with perception delivered by the products incorporates finally the products in the cloud-based design and manufacturing process.

Agent Classes. The entire MAS society is composed of different agent classes that satisfy different sub-goals and reflect the sensing-aggregation-application layer model: event-based sensor acquisition including sensor fusion (Sensing), aggregation and distribution of data, preprocessing of data and information mapping, search of information sources and sinks, information delivery to databases, delivery of sensing, design, and manufacturing information, propagation of new design data to and notification of manufacturing processes, notification of designer, end users, update of models and design parameters. Most of the agents can be transferred in code frames with a size lower than 4kB, and depends on the data pay-load they carry. At run-time, agents are instantiated from these different classes, and agents can change to a subclass behavior depending on current sensing, goals, and their inner state.

### 4.3. Case Study 3: Tailor-Made Shoes—Value Co-Creation Approach with Reactive Rubber 3D Printer

The present case study details a consumer product scenario (shoes and the shoe industry) studied in some detail in the course of a publically-financed project inspired by the needs of a local industry and aiming at the validation of a smart factory concept that incorporates many features of UDE-CBDM as discussed here.

Because of current trends in modern life and longevity, it has become common to wear shoes daily for long periods. Consumers devote great concern to “foot comfort” or “shoes fit feeling” in various life scenes, such as health promotion, rehabilitation, running or other athletics, business use, *etc.* Rubber is an important material used to develop shoes of various types to achieve comfort levels and/or customer satisfaction.Kobe, the birthplace of the rubber industry in Japan, is still the country's largest base of chemical shoes despite damage caused by the Great Hanshin-Awaji Earthquake. Therefore it is important for the Kobe area to realize and maintain the innovative capabilities of the rubber industry to secure its footing in the global competition. The current study’s target is the athletic shoe industry as the first step to validate a smart factory concept with user involvement.

**Figure 11:**
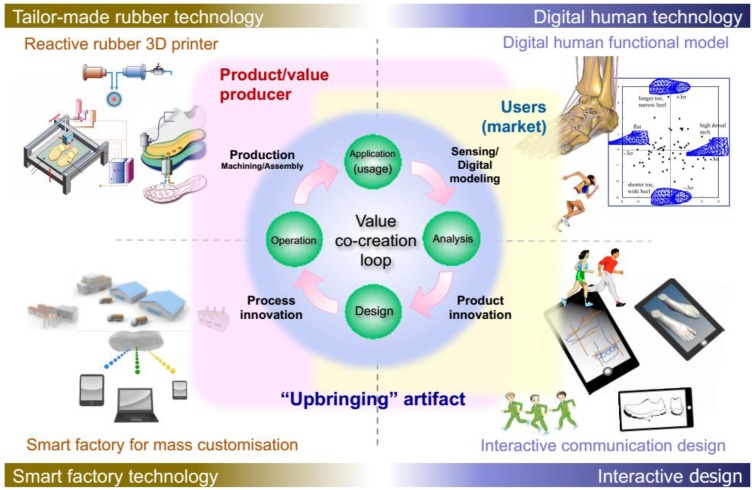
Proposed value co-creation loop.

The value co-creation loop in our project is presented in Figure 11. The loop consists of four major processes: Analysis, Design, Operation and Application.

Digital human technology is applied to conduct comprehensive analyses based on market data. User motion data and biological data are obtained precisely from the user market with sensing devices such as motion capture or CT scan equipment. Then interactive communication design is done based on the analytic data. Communication is realized with several smart devices under an IoT environment. Service applications connect producers and users, who can send attributes or preferences to producers, who provide tailor-made product design services including athletic functions and surface design.

The diversity of products is increased greatly by tailor-made production. This implies that the smart factory technology for mass customization production is operated from the design to operation phase as process innovation. Distributed and autonomous production mechanisms are implemented transparently within the smart factory to correspond with several levels of user satisfaction in an IoT environment. AM has a major role in this concept, as it is mandatory to achieve the required customization levels: We can attempt to invent a new reactive 3D printer for rubber materials to attain tailor-made rubber products in the production phase. Rubber products with functionally graded materials are realized with embedded pressure-sensitive sensors. Consequently, any kind of users’ personal comfort related to shape and function of athletic shoes (*i.e.*, inner/mid/outer soles) is attainable with the reactive rubber 3D printer.

Rubber products can be integrated into the soles of running shoes of professional athletes (lead users). Then the information collected through the sensor under an IoT environment should be applied to shoe design for the more casual runners (semi-lead users), and eventually for mass users in society. The level of each phase in the loop differs according to the loop hierarchy. Design intellectual transformation from lead users to mass users is executed via a three-layered-business system.

The entire project design, particularly the iterative data collection and the tailoring process for customers, involves “service design”. Subconscious user delight is extracted using the proposed continuous interactions amongst users of several kinds and producers in the business system. User centered-innovation is achieved in our project with reactive rubber 3D printing technology.

**Figure 12:**
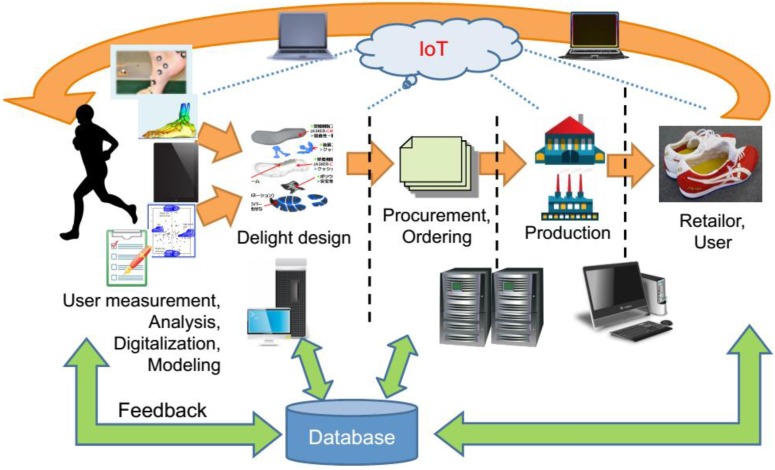
Proposed smart factory structure.

A new smart factory including the concept of “value in use” is realized in this research project. As an example of our development, our smart factory structure for mass customization of the athletic shoe industry is presented in Figure 12.

A smart factory encompasses all business layers in the industrial value chain including consumers with IoT environment. Most software modules are integrated in manufacturing management and control, such as ERP, SCM, MES, and SFA. Then a multi-agent based intelligent CPS system, named a Real-Virtual fusion System [132], is implemented into the smart factory to realize distributed and autonomous factory management and control. Thus apart from the fully-automated cloud-based design and optimization system, which forms the core of the suggested DaaS system, all aspects of theUDE-CBDM concept proposed are reflected in this case study, including the gathering of usage data through lead users. Effectively, this constitutes the definition of a distinct user group, the data gathered by which is intended to support optimization tasks for additional user groups. Contrary to the full UDE-CBDM concept, secondary and ternary user groups will not be drawn on to a similar degree, *i.e.*, no sensor integration is foreseen in their case. Of specific interest, however, is the fact that the smart factory concept does include user feedback, and even indirectly so, through the establishment of communication links between users and producers, potentially on social network level or using similar concepts, and the analysis of this communication.

## 5. Discussion

**Figure 13:**
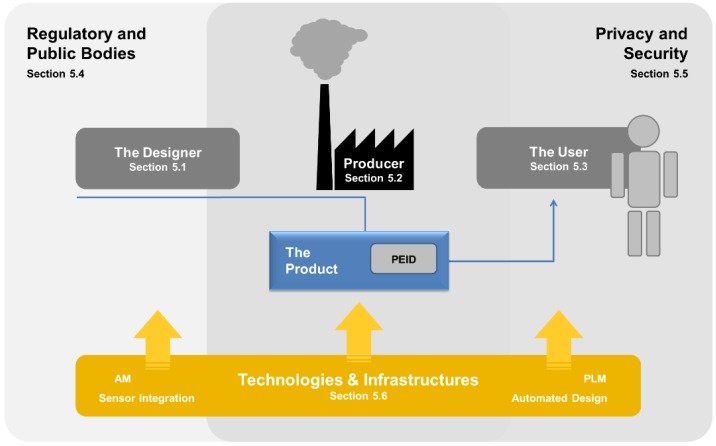
The main stakeholders and perspectives and their relation to each other.

As presented above, the UDE-CBDM scenario defines and demonstrates a trend affecting several stakeholders. In the present section, a critical reflection of assumptions made in this context and their implications is presented using the main stakeholder’s perspectives (*i.e.*, designer, producer, user and regulatory or public bodies) as structuring element. In addition, general challenges related to privacy and security concerns are highlighted. Figure 13 denotes these various points of view, adding technologies and infrastructure as their common foundation. The discussion itself will not be dogmatic in the sense that only a full realization of the UDE-CBDM scenario is considered worth discussing. In practice, several prior, intermediate levels can be envisaged.

### 5.1. Designer’s Perspective

With the notion of products being continuously adapted depending on how they are actually used, designers have to acquire additional skills and knowledge in several different domains. Selected examples are explained in the following.

First, the ability of production to adapt to and thus facilitate variation from product item to product item requires that products are designed with extensive flexibility in mind [133]. A similar development was necessary for the transition from mass production towards mass customization, where the intense use of modular product architectures and parametric design allows customers to select those system configurations that would fit their purpose best [134]. For reason of such characteristics, parametric design has been linked to both simulation and design optimization [135,136] and digital, *i.e.*, additive manufacturing [137]. In principle, however, it remains a way to describe, on different levels, the geometry of a product as function of a set of parameters that can be treated as variables for creating design variants [138]. The present UDE-CBDM approach needs flexibility on a much finer level. Consider a design that is controlled by the ability to withstand certain mechanical loads as main requirement. Typically, information derived from usage data will “refine” these requirements, suggesting limited change to a preceding or to the initial “root design” set to achieve improved performance—e.g., weight-specific strength. Modularity alone will not suffice to fine-tune such a design, specifically if it is already nearing saturation on a generation-to-generation, or rather item-to-item, performance increase curve. Effectively, parametric design needs to be extended to incorporate additional features such as local material properties—provided that these can be accessed by the manufacturing process chosen. AM has this potential: Developments like the so-called Digital Materials approach [139] are currently being commercialized by companies like Stratasys. In this latter case, under the trade name PolyJet, a material jetting technology based on inkjet printing using multiple print heads is employed. Within the boundary conditions of the process in general, this strategy permits voxel-to-voxel change of materials and thus properties, allowing design of materials e.g., with tailored mechanical performance [140,141]. Similarly, process classes like directed energy deposition or 3D printing as example of the binder jetting approach may develop in this direction, as they allow either switching between materials (DED) or controlled modification through additives for which the binder serves as carrier medium [81]. Integrating such local material behavior in the design workflow will afford several changes, including the representation of the product as a CAD model and the translation of the latter to a production oriented format: For this purpose, the standard solution in AM, the STL file format, is not suitable anymore [140]. In terms of simulation-based optimization, established approaches like shape or topology optimization likewise fail to capture the added complexity. Further advancement of methods like Multi-Phase Topology Optimization (MPTO) introduced by Burblies *et al.* can help close this gap: The methods provides optimum distributions of porosity levels within mechanically loaded components [122] and has been demonstrated to be transferable to AM-based implementations. The envisaged DaaS toolset, in its optimization and CAD modelling capabilities as well as in its output to the manufacturing system, will have to apply such features, and allow their modification in response to usage data input.

Secondly, with an increasing flexibility in design, an extensive knowledge of the dependencies among different system components is needed. Making one part flexible might lead to immediate or long-term consequences for related elements of the whole system. The same holds true for solely component-centered optimization which ignores interdependencies within the overall system and may thus lead to performance reduction or even premature failure on system level.

Thirdly, a critical aspect of adaptive products is that adaptations must meet high quality standards at any moment of the product life. In contrast to biological systems, that perform a trial-and-error approach of which death is just a necessary and accepted concept, technical systems must avoid costly failures. In order to guarantee such levels of quality, testing procedures have to be applied during product design and development. However, physical testing fails to fulfil the basic demands of our suggested approach for two reasons: Since it requires production of prototypes and availability of test facilities it contradicts the notion of individually optimized products on a level of principle, and it eliminates much of the advantage in lead time reduction that AM as production method offers. Furthermore, it would limit the choice of possible production sites to those that have the right test equipment at hand, and would thus cancel out the envisaged decentralized production option. For these reasons, affordable personalized and individually optimized products as foreseen in this paper must make extensive, if not exclusive, use of virtual testing methods and environments.

Fourthly, taking usage information into account during design decisions requires knowledge in representation and interpretation of the information. Representation is bound to data analysis techniques ranging from basic statistics to complex data mining approaches. In using the gathered data, distinctions have to be made regarding the level on which optimization is implemented. The following main categories can be identified:

Optimization on individual/item level: A follow-up product serving the same purpose for the same user can be optimized to meet an adapted, personalized set of requirements.

Optimization on subgroup level: Collection of PLM data may reveal certain recurring types of usage, allowing identification of and thus product optimization for certain user subgroups. As in the previous case, this implies that additional, defined sets of requirements can be formulated on subgroup level that deviate from and/or add to the generalized set.

Optimization on population level: On a general level, collection of PLM data will establish a major step forward in terms of usage patterns and thus help to focus and/or boil down the set of requirements all individuals/items of a product (on product definition in this context, see statements below) have to conform to.

Distinction between these levels needs to be reflected in the data analysis techniques employed. An additional aspect worth mentioning in this context is the fact that in a scenario with item-level optimization, all data will be gathered by slightly deviating individual products. Thus it is necessary to distinguish the lessons to be learned from the usage data. One part concerns lessons about the use case (e.g., involved actors and product functions), and a second part concerns those about the actual product item that gathered the respective data set. It is furthermore important to derive rules that allow combination of data originating from different product items, and thus different expressions of a common “root design”. Capabilities of this kind would be associated to the interfacing and mapping element specifically highlighted in Figure 8.

More generally speaking, the interpretation of information is a matter of human experience and understanding of context. Advances in data analytics techniques created a new profession, the data scientist [142]. In order to learn from usage data, designers must collaborate with data scientists to find appropriate answers for design problems within the data. Typical examples are the search for new requirements or the validation of existing requirements typically documented before starting to create or alter a product’s design. Besides technical and collaboration challenges related to the analysis of usage information, it is also necessary for designers to understand the limitations of data analytics. An important concept that must be considered in this respect is information quality [143]. Any piece of usage information is created within a certain context and used for one or more specific purposes. In the case of sensor data, a specific class and make of sensor is operated to acquire the raw data. The context of each sensor (e.g., whether it was calibrated or not) is needed to correctly interpret the resulting data. Without this information, designers and data scientists must be careful concluding on the data. In addition, the understanding of complex usage patterns from sensor data may require complementary information, such as user-authored information in discussion forums, social networking services, maintenance reports and product reviews. Besides that, information from complementarily used products should be taken into account to gain a better understanding of user problems. The improvement of shoes, for instance, might need to take data from a backpack (e.g., load during movement) or umbrella into account (e.g., local weather). Such complementary information sources add to the context of a specific design decision and may thus reduce uncertainty—though here, too, quality of information has to be taken into account.

All the above needs to be considered beforehand and designed into any future system capable of automating the design optimization process, thus taking central decisions in this field out of the hands of human designers. Automated design must evolve to deliver valid solutions fast and in a directly applicable manner based on the growth and evolution of the available usage data. This implies first the connection of the relevant tools applied in the design process to these data, secondly the feeding of these with knowledge and rules on AM processes. Today, the latter is still seen as a major obstacle to the distribution of AM technologies even in a conventional design and development process, since many of the specific rules of “design for AM” have not been formulated, let alone spread widely yet.

### 5.2. Producer’s Perspective

Before discussing the producer’s perspective in the following, the difference from the designer’s perspective has to be elaborated. In the described scenario, this differentiation is more fluid than is known from the related traditional structures in industry. In UDE-CBDM, design and manufacturing is a continuous effort, which requires exchange of information and transparency throughout the value-chain. Thinking this even further, the “producer” might even be the user him/her-self, employing decentralized AM capabilities at home or in the local Maker-Space. In such a case, the “traditional” producers responsibilities may be reduced to supplying the material (if that is the case at all), or to serving as an intermediary supplying the parameters/specification for the AM process at the users site—or his role might even become completely obsolete.

However, this extreme example is still far from reality. Industrial AM equipment is still rather costly, not only regarding the tools themselves but also the service contracts required to keep the system in a working condition. Therefore, the role of the producer is valid and an important component of the value chain in this vision. The re-shuffling of the latter will also open up new business opportunities both for the producer, and for third parties that have access to the usage data that product and product groups supply. Figure 7 illustrates some of the potential connections in this respect.

**Figure 14:**
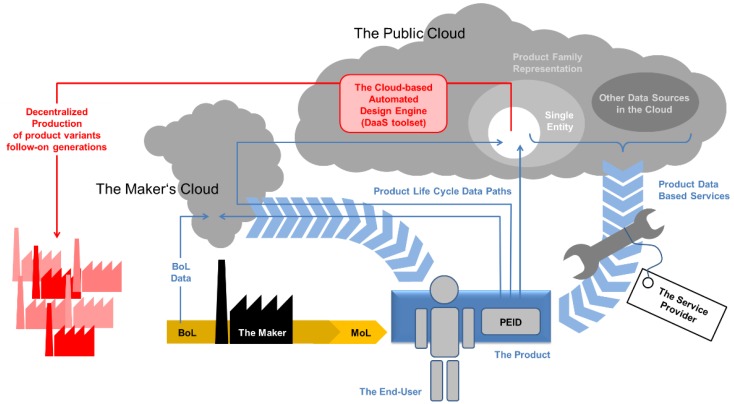
An overview of data flows between stakeholders integrating the cloud and highlighting possibilities of cloud-based product-related services.

The basic assumption is that usage data gathered by the product can either stay within the maker’s realm, where it is accompanied by data associated with the manufacturing of the part, *i.e.*, BoL data, or it can be passed on into a “public” cloud, naturally assuming that all necessary steps for ensuring security and privacy (see Section 5.5) have been taken. The availability of such data facilitates several potential product-related services, which may be offered by the maker or by third parties if these have legal access to the data: The fundamental business case for the original producer of a part could thus be the provision of a (personalized or non-personalized) optimization service to the customer. Further product-related services may include maintenance, which could be handled by the maker, or left to third parties. In the latter case, depending on the legal situation in that respect, the producer might enter into a business relationship with the services provider, granting him access to standard or extended product-related data. In parallel, the service provider could use other cloud-based resources to add to the product-specific data and thus enhance or extend his services. A special case is the full-fledged scenario assuming an automated design engine, the DaaS toolset, that creates the subsequent product generation automatically (note that the optimization service could also be envisaged following a conventional design approach), once again making use of available data on the preceding generations. Here, redesign could be a service offered via the cloud, with not so much the primary, but potentially secondary producers acquiring access to the redesign and optimization tool, which might be offered by a software developer. Since the original maker would not be involved here, Beginning of Life (BoL) data as a additional source of information would probably be lacking, however, for the new product entity, its new, originally secondary, producer would assume the role of primary maker, with all the facets this entails according to Figure 14.

Being responsible for transforming the virtual design in a physical product, the producer’s responsibility includes ensuring that the manufacturing processes deliver the required quality as it is the case today. Due to the flexibility of AM and the availability of the design in the required format, the impact of the producers’ expertise will shift slightly towards supporting the designers from an early stage on. This support includes but is not limited to: Design for Manufacturability (DfM), choice of available material, dimensioning (based on available equipment).

Besides, defining the new role of the producer requires answering a multitude of open questions. Decentralization of production, including item-to-item adaptation of design will require formulations that clearly define responsibilities along this process chain: If a product fails on item-level, who is to blame? Will it always be possible to associate the cause of failure with either root design, collection of usage data, interpretation of it, transfer of data (cyber security aspects), product optimization or production? In this context, we need to take into account that it might not be practically feasible to establish an answer on a case-by-case level. Thus there might be a need for generalized rules, which would mean that the relationship between, for instance, designer, manufacturer and service provider may have to be re-thought and cast into a working formalism, be it on a contract or licensing level, or another suitable mode.

Finally, the producer, bound by current regulations, may also find him-/herself responsible for the recycling of the products after they reach the end of their service life. As each product is unique in the most advanced scenario, this might add additional challenges. These challenges include dismantling in case sensors are implemented (at different, item-specific locations), hazardous materials, precious metals and others.

### 5.3. User’s Perspective

For a user, our scenario has several benefits in store, not the least among them the chance to obtain optimized follow-up products adapted to his personal needs. There is, however, no rose without thorns, and in the present case, to realize these advantages, the user may have to become an active partner of the producer. Currently this must seem an uncommon relationship thinking of everyday goods like shoes, and it must be taken into account in this context that even if the individual task may seem negligible from a single product perspective, it will accumulate the more candidate products the user chooses to maintain in this same way. The actual tasks the user might be entrusted with may include the read-out of data, the added, personal evaluation of product performance and performance change, as well as maintenance tasks that are aimed at ensuring the quality of data to stay on a constant, high level throughout the product’s lifetime.

A side aspect of the implied personalization of products may be the impossibility of allowing them to be used by other people aside from the owner. Both in private life and in a sharing or collaborative consumption economy, which currently seems to evolve in several fields, this may prove a restriction felt severely by many users.

Finally, the big issues when it comes to usage data are privacy and ownership of data: These will be treated in more detail in Section 5.5, since they touch upon the realm of users and other stakeholders alike.

Considered along the above lines, the user is the end user or customer that will directly utilize the final product—wear the shoes, if we refer to case study 3, or the smart implant, if we consider case study 1. In practice, the interface between this user and the cloud-based services that lead to his personal product might often be provided by a dedicated service provider. For both, the customer or end-user and for any intermediary, it is very important to gain an idea of the reliability of the manufacturing chain behind the interface to service provider (as potential intermediary), or the cloud-based services themselves. In a conventional production scenario, the end-user would base his trust e.g., on the reputation of a brand, *i.e.*, an OEM. In CBDM in general, and thus also in its UDE-CBDM variant, the manufacturing chain is much more susceptible to change, as the model implies choice of manufacturing opportunities based on factors like availability and price raised almost in real time at the instant an order for a product is placed. Thus instead of fixed, well established supplier relations, a constant reconfiguration of the chain is possible from single order to another single order. In such an environment, subjectively building up trust is rendered difficult if not impossible. In consequence, as Li *et al.* point out, some formal, more objective mechanism for evaluation of trustworthiness needs to be established [144].

### 5.4. Regulatory or Public Bodies

In its practical implementation, the final consequence of our approach will be a certain loss of meaning for the term product series. Many of the above statements imply that products can be identified on population level as belonging together through a set of measurable—and this means measurable on the product—parameters that for this population take on the same value. This is certainly true in conventional manufacturing, when e.g., the same die is used to cast a series of wheels. However, according to our present approach, such commonalities are lost because in the extreme case, a redesign process is initiated prior to the manufacturing of each individual and based on the usage data available at that specific moment in time: For a product made a few minutes later, the data base may already have changed. What constitutes a product in this world could then only be a generalized description of purpose, *i.e.*, of the application scenario, and a set of equally general common requirements. Neither of these would suffice to actually explain all the specific properties of an individual product, because these would be based partly on the general requirements, and partly on individual- or subgroup-related ones. It would thus only be possible to speak of a product in the broader sense of a group of objects built to meet a common class of general requirements, and having a common “root design”.

Considering the practical implications of the above conjecture, it is obvious that under such circumstances, the concept of type approval and homologation cannot be maintained. On the other hand, it is similarly obvious that it is impossible for economic reasons to seek approval on a single item level.

Here, a solution comes to mind which has already been discussed in terms of its role in the design process in Section 5.1: If physical testing is out of question, virtual testing must take its place. This, however, does not eliminate the practical issue that not every individual product entity can be virtually tested by a certifying authority. Thus wherever certification of products is an issue, this means that the virtual test procedures themselves must first be acknowledged and certified, to guarantee their correct application, and to allow the results to be accepted as proof of the product’s adherence to the relevant rules and standards. Similarly, it might be possible to evaluate the optimization procedures that govern entity-to-entity product evolution. In this case, a conventional certification of a “root design” could be implemented and “measures of change” developed: Their limiting values would then define a corridor the violation of which by product characteristics would set the mark for recertification, turning the then-current product design stage into the new root design with respect to certification. To make such a process calculable for the producer, predictive tools might be needed allowing an estimate of levels of change to be expected within a given period of time, in order to identify the tipping points in advance and prepare for the recertification effort in time.

The fundamental assumption in the discussion above is that certification authorities and regulatory bodies have to deal with a single, combined designer and producer. Needless to say, the issue becomes more complex once the elements of decentralized production and cloud-based optimization are added. Design evolutions would then occur independent of the “root designer”, which would raise additional liability issues. Once again a step towards a solution could be to certify the methods and their use, and not the product, or rather product state. However, as already mentioned in Section 4.2, even if this approach proved possible, liability issues along the chain or rather network of designer, initial producer, optimizer, secondary producer and customer would still need to be clarified, and accepted, and potentially globally enforced rules in this respect laid down.

### 5.5. Privacy and Security

Another general challenge beyond our specific scenario is the question of data ownership and privacy, which has been introduced already in Section 4.3. Usage data is by definition generated when the product is used, and by the user. Our model assumes, for example, that the original designer of the product, or rather those group that provides the optimization service, has full access to the collected data, or at least the information derived from it. This access may or may not include additional information about the user of a specific product entity, again with a possible differentiation in personal/individual or user group data. Access to this data is likely to allow its evaluator to derive several conclusions on user or user group that go far beyond the needs for product optimization. Thus the user has to be convinced that he/she should share his/her data with the manufacturer/designer—either for his personal (item-level approach), or for the mutual benefit of broader user communities (subgroup and/or population level approach). This entails legal issues, like misuse of sensitive data *etc.* However, as this is a strongly discussed topic today, there is a large chance that this issue will be solved one way or another within the foreseeable future. In any case, it is clear that a legal framework has to be created that clearly define the involved parties’ rights as well as their limitations.

Besides privacy, cyber security is becoming more and more of a concern both in the area of networked smart systems and objects (from cars to IoT) and in that of Additive Manufacturing. Here, we combine both aspects. New issues include questions such as product identification and protection against plagiarism, but also the issue of assuring that data has not been corrupted in some way or another on its path from designer to producer, with potentially critical consequences for product safety, to give but one example. Consistency of data thus needs to be ensured.

An increasing degree and complexity of networking and information exchange between a large number of computers, mobile devices, sensor networks, individual smart sensors, products and machines induce new challenges in the handling of privacy of data, privacy of users, authorization, and authentication in a mostly anonymous Internet-of-Things (IoT) domain. New concepts and methodologies are required for binding and granting access rights to data, creating access roles based on application rather than on user level, traditionally managed by higher communication layers like WEB servers or databases providing account services. Considering consumer products that deliver lifecycle data, the users and their location are mainly unknown. By enabling usage data delivery from products to manufacturers these anonymous users get a virtual identity encapsulating private user data, for example, their geographic location (that can be accurately determined by network services and fusing of other data), the usage behavior, and so on. In the proposed design and manufacturing clouds, which are integrated in the vulnerable Internet, Cyber Attacks can significantly disturb the processes or spy sensitive or confidential data. If sensor, product, and user data is autonomously transferred, for example, by mobile agents, the data become self-contained units that can be processed on any platform, ranging from embedded systems up to servers. It is relatively easy for intruders to include Man-of-the-Middle attacks to retrieve and spy secured data, and in service-orientated processing environments Denial-of-Service (DoS) attacks can prevent an overall system operation. Currently the security issues concerning stealing data or attacking services are not addressed properly in the IoT community. Security issues are related to the user (data producer) and service provider (data consumer) side, though both sides have different goals and constraints in mind.

Looking at cyber security specifically from a producer’s perspective, as Lu *et al.* point out, it must be taken into account that the importance of intangible knowledge assets, in other words, of data, for organizational success in industry is clearly on the rise, while at the same time, the accessibility of data and thus vulnerability of the respective assets is increasing with any bit of data that is mirrored in the cloud. The dilemma for the producer is between the wish to raise the potential of cloud-based services and keep track of his competitors in that respect, and the risks that goes with moving business into the cloud. Consequently, dedicated strategies for ensuring cyber security are mandatory [145].

## 6. Conclusions and Outlook

In the present work, we have outlined the characteristics of a new manufacturing paradigm designated as Usage Data-Enhanced Cloud-based Design and Manufacturing (UDE-CBDM). We have explained why we believe

- usage data collection and analysis, facilitated by
- sensor integration in
- additive manufacturing and fed into
- automated design processes

may develop into the main foundations of our approach, and along which paths this needs to happen. We have furthermore discussed the role of cloud-bases services in this context, and the potential these offer, e.g., in terms of new business models associated to the suggested alternative organization of the product development and manufacturing process. In considering the above research domains, we notice that many of our concept’s prerequisites are either there or under development, and that to a significant degree, the challenge of realizing UDE-CBDM translates into the challenge of correctly arranging and interfacing these building blocks.

Within the PLM domain, new frameworks and methodologies are surfacing at ever-increasing rate. Especially item-level based approaches utilizing the potential of usage information are rather popular [11,146]. Sensors neatly integrated in the products in question are the initial prerequisite without which the manufacturing paradigm we have outlined above could not fly. Similarly, applying this information for product optimization needs a production process characterized by highest flexibility and lowest lead times. From the aforesaid, the need for AM in this context and its crucial role on a technological level is obvious: Continuous redesign and individualized production is only possibly if adaptation to design changes is next to instantaneous and investment costs are not linked to an individual product design, as is the case when moulds and tooling are required. AM, and at present only AM, can successfully relinquish these obstacles. Currently, however, the 3D printer that generates an engineering component with integrated sensor systems in a single step or setup still has to be designed, even though its predecessors are available on lab-scale, as is documented e.g., by Espalin *et al.* [80]. Neat integration of sensors and sensor systems thus needs to be further advanced to the level of material-integrated intelligent systems and against the background of AM-based production.

The drive that AM adds to the vision is best understood when comparing it to earlier models envisaging an evolutionary approach to product design: A good example in this respect is the gentelligent design approach formulated by Lachmayer *et al.* [12,147]: Here, evolution is considered on a generation-to-generation level, with continuity upheld mainly with respect to collection of PLM data. This strategy thus represents a cut-out of what has been designated the Autogenetic Design Theory (ADT) by Vajna *et al.* [148]: “Evolution means gradual development, permanent adaptation, and continuous optimization towards an aim that may also change itself during the evolution […]. Additionally, dynamic starting conditions, boundary conditions, and constraints influence natural evolution, which is exactly the same as in any design” [148]. The objective of ADT is to understand the ruling principles behind this scenario from an engineering point of view and implement them in product development processes to facilitate a comparable, continuous evolution in product design [148].

It is worth considering, at this stage, that the present concept, when seen as a manifestation of ADT, is in some ways more powerful than its biological equivalent, and may thus approach an envisaged optimum stage significantly faster. In biological evolution, the selector is life itself, and thus the underlying timescale is that of life and reproduction: The ability of a new individual to survive and multiply in its environment provides directions for changes to become dominant in future generations. Variation of individuals is achieved by mutation, but even more so by more or less controlled combination of the genetic background of two parents. Thus success stories of survival from the top down to the root of the parents’ family trees are covered, while other trees rooted in the same forest are neglected. In contrast, in our vision, redesign prior to production of a new item can in principle draw an all the data available at that moment in time—data from all the trees in this and other forests. In practice, this means that the scope of usage data will rapidly outgrow the possible experience of a single individual. With availability of information accelerated by accumulation from several sources, product optimization may progress faster, as compliance and performance improvements will be verified in the virtual rather than the real world, through evolved simulation and optimization tools as part of the suggested DaaS system. Besides, if customer or customer group are known, and thus a detailing of usage patterns is available, optimization can be customized by basing it on a prognosis of this specific item’s most probable lifecycle.

The implication that the product development process will never reach an end will afford investment in automation of the design process. The expertise required in product development as a business case may thus shift to different foci. This shift will require moving towards a higher-level understanding of formal product development processes which in their practical implementation on product level could then be performed automatically. A major challenge in this respect is the mapping between usage data on the one hand to design parameters on the other. Most likely this will require a new look at parametric design, which has to be extended beyond geometric feature description in order to allow the fine-tuning of design solutions our concept rests on. What comes in here, too, is the quality of the available data. Usage data can be rather diverse, ranging from numerical sensor data to complex user feedback in an online forum. The overall system needs to be robust enough to handle this diversity and the added complexity of varying information quality within a category, as e.g., caused by sensor malfunction and an associated lack of data.

Thus our present conclusion is that though many of its elements are available today on a sufficiently advanced level to realize several of its major aspects, our paradigm still bears the basic characteristics of a vision: Some technological issues that have to be addressed we have highlighted in the above. However, there are other, less technical challenges which need to be handled, too. These are touching aspects like privacy, cyber security, ownership of data and product certification, which have to be considered on a legislative and regulatory level, too. Product certification is a good example in this respect, as it links technological with regulatory issues: We all rely on the safety of the products we are using—but what if these are not accessible to physical evaluation prior to the product’s market introduction? To answer this question, implementations of cloud-based continuous product optimization will have to establish documented, traceable and reproducible virtual testing procedures which make sure that even a product item that is customized to its unique user is in fact safe to the required degree—and needless to say, the applicability of these procedures must be laid down in the relevant regulations, meaning that they, too, need to be certified in some way.

With so many issues still open, why embark on the voyage we propose? In high-wage regions like the USA or Europe, AM is seen as a vehicle that might help relocate production to places that today have lost their industrial backbone to global competition: Surely, additive manufacturing in itself has the potential to reshuffle global value chains. However, by presenting our concept, we believe we have shown that to make best use of this potential, combining the power of AM, which is mainly rooted in its flexibility, with several other emerging trends in gathering, managing and analyzing large amounts of data can lead to even more disruptive paradigm shifts in production engineering. It is our conviction that any stakeholder willing to maintain or even strengthen his footing in the manufacturing domain must invest in understanding both the challenges and the potentials linked to cloud-based manufacturing in all its possible variants. With the complementary technologies that fuel our vision currently evolving at similar, rapid pace, change may come fast, and who would want to be the dinosaur unable to adapt in time?

## Abbreviations

ABM	Agent-Based Manufacturing
ADT	Autogenetic Design Theory
AM	Additive Manufacturing
ASTM	American Society for Testing and Materials
BoL	Beginning of Life
CBDM	Cloud-Based Design and Manufacturing
CBM	Cloud-Based Manufacturing
CMfg	Cloud Manufacturing
DaaS	Design as a Service
DEM	Discrete Element Method
DLF	Directed Light Fabrication
DLP	Digital Light Processing
DMD	Direct Metal Deposition
DMLS	Direct Metal Laser Sintering
EBAM	Electron Beam Additive Manufacturing
EoL	End of Life
FDM	Fused Deposition Modeling
FGM	Functionally Graded Materials
FEF	Freeze-Form Extrusion Fabrication
HaaS	Hardware-as-a-Service
IaaS	Infrastructure-as-a-Service
LCE	Life Cycle Engineering
LMD	Laser Metal Deposition
LLM	Layer-Laminated Manufacturing
LOM	Laminated Object Manufacturing
MID	Moulded Interconnected Device
MJM	Multi-Jet Modeling
MJS	Multiphase Jet Solidification
MoL	Middle of Life
MS	Mask Sintering
NIST	National Institute of Standards and Technology
OEM	Original Equipment Manufacturer
PaaS	Platform-as-a-Service
PDB	Plate Diffusion Brazing
PEID	Product-embedded information device
PJM	Polyjet Modeling
PLM	Product Life Cycle Management
SHM	Structural Health Monitoring
SaaS	Software-as-a-Service
SL/SLA	Stereolithography
SLM	Seletive Laser Melting
SLS	Selective Laser Sintering
SMD	Surface Mounted Device
SMT	Surface Mount Technology
STL	Stereo Lithography, alternatively Standard Tesselation Language, a file format for representing model data for various AM systems
UAM	Ultrasonic Additive Manufacturing
UC	Ultrasonic Consolidation
UV	ultra-violett
WBM	Web-Based Manufacturing
